# Coordinated epigenetic remodelling of transcriptional networks occurs during early breast carcinogenesis

**DOI:** 10.1186/s13148-015-0086-0

**Published:** 2015-05-01

**Authors:** Warwick J Locke, Elena Zotenko, Clare Stirzaker, Mark D Robinson, Rebecca A Hinshelwood, Andrew Stone, Roger R Reddel, Lily I Huschtscha, Susan J Clark

**Affiliations:** Epigenetic Research Laboratory, Genomics and Epigenetic Division, The Garvan Institute of Medical Research, 384 Victoria Street, Darlinghurst, NSW 2010 Australia; St. Vincent’s Clinical School, Faculty of Medicine, University of New South Wales, Level 5 deLacy Building, St Vincent’s Hospital, Victoria Street, Darlinghurst, NSW 2010 Australia; Swiss Institute of Bioinformatics, University of Zurich, Zurich, and Institute of Molecular Life Sciences, University of Zurich, Winterthurerstrasse 190, Zurich, CH-8057 Switzerland; Sydney West Cancer Trials Centre, Crown Princess Mary Cancer Centre Westmead, Westmead Hospital, Hawkesbury Road, Westmead, NSW 2145 Australia; Cancer Research Unit, Children’s Medical Research Institute, 2145 Hawkesbury Road, Westmead, NSW 2145 Australia; Sydney Medical School, University of Sydney, Fisher Road, Sydney, NSW 2006 Australia

**Keywords:** DNA methylation, Methylome, Epigenetics, Basal breast cancer, Epigenome sequencing, Biomarker

## Abstract

**Background:**

Dysregulation of the epigenome is a common event in malignancy; however, deciphering the earliest cancer-associated epigenetic events remains a challenge. Cancer epigenome studies to date have primarily utilised cancer cell lines or clinical samples, where it is difficult to identify the initial epigenetic lesions from those that occur over time. Here, we analysed the epigenome of human mammary epithelial cells (HMEC) and a matched variant cell population (vHMEC) that have spontaneously escaped senescence and undergone partial carcinogenic transformation. Using this model of basal-like breast carcinogenesis, we provide striking new insights into the very first epigenetic changes that occur during the initial stages of malignancy.

**Results:**

The first phase of malignancy is defined by coordinated changes in the epigenome. At the chromatin level, this is embodied in long-range epigenetic deregulation, which involves the concomitant but atypical acquisition or loss of active and repressive histone modifications across large regional blocks. Changes in DNA methylation also occurs in a highly coordinated manner. We identified differentially methylated regions (DMRs) in the very earliest passages of vHMECs. Notably, we find that differential methylation targets loci regulated by key transcription factors including p53, AHR and E2F family members suggesting that epigenetic deregulation of transcription factor binding is a key event in breast carcinogenesis. Interestingly, DMRs identified in vHMEC are extensively methylated in breast cancer, with hypermethylation frequently encroaching into neighbouring regions. A subset of vHMEC DMRs exhibited a strong basal-like cancer specific hypermethylation.

**Conclusions:**

Here, we generated epigenome-wide maps of the earliest phase of breast malignancy and show long-range epigenetic deregulation and coordinated DNA hypermethylation targets loci regulated by key transcription factors. These findings support a model where induction of breast cancer occurs through epigenetic disruption of transcription factor binding leading to deregulation of cancer-associated transcriptional networks. With their stability and very early occurrence, vHMECs hypermethylated loci could serve as excellent biomarkers for the initial detection of basal breast cancer.

**Electronic supplementary material:**

The online version of this article (doi:10.1186/s13148-015-0086-0) contains supplementary material, which is available to authorized users.

## Background

The epigenetic control of gene expression plays a critical role in the development of normal cells and is extensively deregulated in all cancers, including breast cancer (reviewed in Baylin and Jones [1]). Hypermethylation frequently occurs in high-density clusters termed CpG islands and is often associated with gene promoters and transcriptional repression in cancer, whereas decreased methylation at regulatory elements is associated with gene activation [1,2]. Alterations in DNA methylation contribute to carcinogenesis via a variety of mechanisms including the silencing of tumour suppressors [1,2] and reactivation of transposons [3], genomic instability [4,5] and blocking differentiation [6]. Furthermore, the cancer DNA methylation profile can be associated with specific molecular subtypes [7-9], various clinicopathological parameters (reviewed in Locke and Clark [10]) and response to endocrine therapy [11,12].

While epigenetic dysregulation in cancer is well established, the timing and extent of epigenetic change during carcinogenesis remains largely unknown. Most studies utilise comparisons of cancer cell lines or cancer tissue samples with normal tissue to identify cancer-associated epigenetic aberrations that occur during tumour development. However, these studies cannot identify the early events associated with epigenetic remodelling during transformation. To address this issue, we utilised the human mammary epithelial cell (HMEC) model system [10,13]. HMECs are derived from normal breast tissue removed from healthy women during breast reduction mammoplasty [14]. In culture HMEC undergo two distinct phases of growth separated by a temporary period of growth arrest similar to senescence, termed ‘selection’. Cells from the two growth phases are termed HMEC prior to selection and then, after overcoming senescence barriers during selection, are termed variant HMEC (vHMEC) during the second growth phase [15,16]. When compared to HMECs, vHMECs exhibit multiple cancer-associated gene expression and epigenetic changes and are considered to represent a partially transformed pre-malignant breast cell (reviewed in Locke and Clark [10] and Hinshelwood and Clark [13]). Such changes include silencing of the p16^ink4a^ tumour suppressor associated with promoter hypermethylation [15,17-19], silencing of the transforming growth factor beta pathway via acquisition of repressive chromatin [20] and increased expression of the cancer-associated chromatin methyltransferase *EZH2* [20]. Due to its extended lifespan and multiple cancer-associated expression changes, the HMEC system provides a model of partial carcinogenic transformation from normal to pre-malignancy. Therefore, the HMEC system is an ideal tool for the identification of the first epigenomic events occurring during early breast carcinogenesis.

In order to understand the role of epigenomic deregulation in breast carcinogenesis, we performed detailed expression, DNA methylation and chromatin modification profiling of a set of HMECs and isogenic vHMECs. We show that epigenomic aberrations in key regulatory pathways and across domains occur during the very earliest stages of breast carcinogenesis. Furthermore, comparison to The Cancer Genome Atlas BReast invasive CArcinoma (TCGA-BRCA) cohort demonstrates that the methylation aberrations we identified in vHMEC are common in basal-like breast tumours suggesting that epigenetic lesions occurring early in carcinogenesis are derived by similar reprogramming events.

## Results

### vHMEC is a model of early basal-like breast carcinogenesis

**Figure 1:**
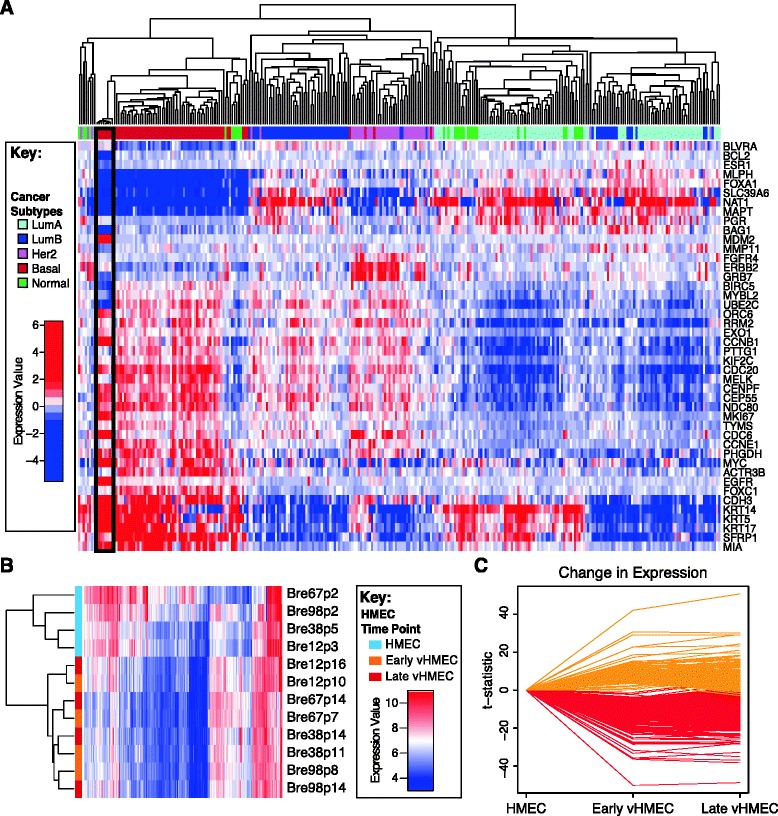
Summary of gene expression changes in vHMEC. **(A)** Hierarchical clustering of the PAM50 expression profile of vHMEC and a breast cancer cohort [GEO:GSE2034] classifies vHMEC (black box) into the basal-like molecular subtype of breast cancer. **(B)** The expression profile of differentially expressed genes in HMEC (light blue) and early and late vHMEC (orange and red, respectively) clusters samples into separate HMEC and vHMEC clusters. **(C)** The *t*-statistic of genes that gain (orange) or lose expression (red) in vHMEC is stable in early and late vHMEC indicating the vHMEC expression pattern is stable even after extended time in culture.

To gain a more detailed understanding of the early epigenetic changes that occur in the first stages of carcinogenesis, we performed epigenome-wide profiling (gene expression, DNA methylation and chromatin modifications [GEO:GSE58882]) of four isogenic HMEC/vHMEC lines (Bre12, Bre38, Bre67 and Bre98). Given their basal culture conditions [14], it is likely that vHMECs resemble the basal-like molecular subtype of breast cancer. To confirm this, we first classified the vHMEC lines into the intrinsic molecular subtypes of breast cancer using Affymetrix GeneChip expression data with the PAM50 classifier [21]. As highlighted by Sorlie and colleagues [22], it is important that expression array data are gene-centred in addition to standard normalisation procedures prior to PAM50 classification. To ensure our findings were reproducible, we performed the gene-centred analysis with two independent publicly available datasets ([GEO:GSE2034] [23] and [GEO:GSE3494] [24]). After clustering, we found that the vHMEC lines from all four donors classified into the basal-like breast cancer subtype in both data sets, supporting the use of these cells as a model to study breast cancer (Figure 1A [GEO:GSE2034] and Additional file 1: Figure S1 [GEO:GSE3494]).

Next we aimed to identify the gene expression changes that potentially drive the earliest steps in development of breast cancer and/or basal breast cancer. First, we performed multi-dimensional scaling (MDS) analysis on the 500 most variably expressed genes across all donors and time points (Additional file 1: Figure S2A) which separated HMEC and vHMEC into two distinct clusters on dimension 1, indicating that selection (that is, the escape from senescence) is the largest source of variation in our dataset rather than inter-individual variation or extended time in culture.

Subsequent limma analysis identified 2,121 and 1,972 genes differentially expressed in early and late vHMEC, respectively, when compared to HMEC (adjusted *P* < 0.05, Figure 1B). Validation of differential expression was performed on a subset of eight genes by quantitative polymerase chain reaction (qPCR) and exhibited high correlation with array profiling (Additional file 1: Figure S2B). The expression level of these approximately 2,000 differentially expressed genes was consistent across both the early and late vHMEC time points (Figure 1C), further indicating that the initial escape from senescence (selection) is the greatest contributor to differential gene expression. Given the high similarity between the vHMEC time points, further expression analyses were performed on the early vHMEC time point only.

### Deregulation of polycomb, MYC and the p53 pathway drive early carcinogenesis

**Figure 2:**
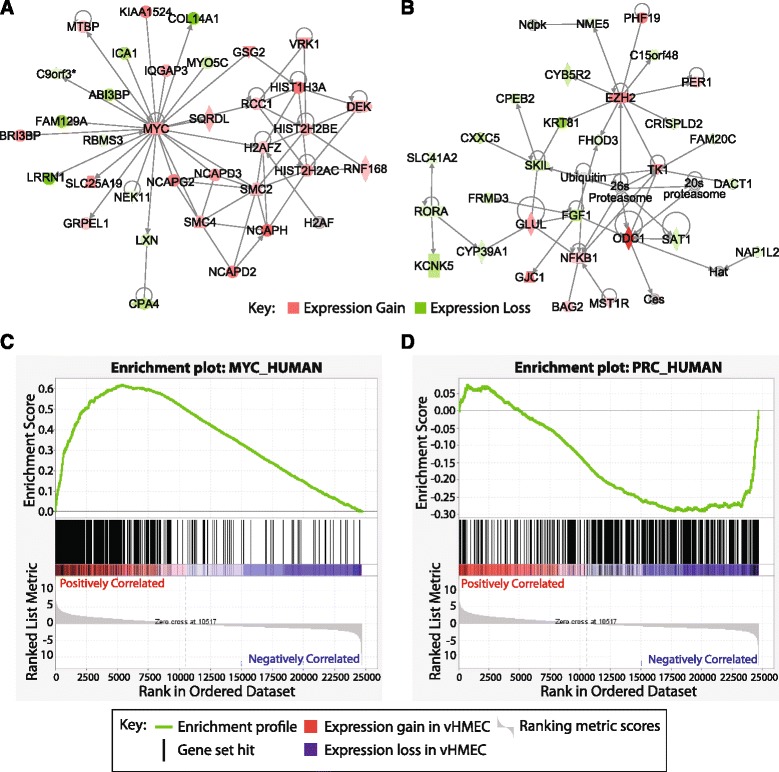
Ingenuity pathway analysis (IPA) identifies deregulation of *MYC* and *EZH2*/polycomb in vHMEC. **(A)** The two most significant gene expression pathways identified by IPA involve deregulation of the proto-oncogene *MYC* and **(B)** the epigenetic regulator and member of the polycomb family, *EZH2* (shading represents *t* statistic). **(C)** Gene set enrichment analysis demonstrates up-regulation of *MYC* targets and **(D)** down-regulation of polycomb target genes in vHMEC in a pattern identical to that reported in cancer.

To understand the impact of gene expression change on the biology of vHMEC and its ability to overcome senescence, we performed Ingenuity Pathway Analysis (IPA). IPA identified that genes differentially expressed in vHMEC were significantly enriched for functions related to cancer (*P* < < 0.001, Additional file 1: Figure S2C), including cellular growth and proliferation, cellular movement, cell cycle and, DNA replication, recombination and repair (*P* < < 0.001). In addition, genes associated with development were enriched, indicating a role for development-related genes in the vHMEC’s escape from senescence. The two most significant pathways identified by IPA contained known breast oncogenes, specifically *MYC* (Figure 2B) and the histone methyltransferase *EZH2* (Figure 2B). IPA transcription factor (TF) analysis predicted activation of both *MYC* and *EZH2* in vHMEC (*P* = 1.33 × 10^−13^ and 2.12 × 10^−03^, respectively, Additional file 2: Table S1). In addition to the identification of gene expression pathways and enrichment of biological functions, IPA can predict alterations in the apparent activity of transcription factors (TF). IPA TF analysis predicted activation of *EZH2* and *MYC*, in agreement with their increased expression levels, as well as several other cancer-associated TFs. These included activation of the NF-κB (including pathway members *NFKB1* and *RELA*), and E2F (including pathway members *E2F1, E2F2* and *E2F3*) signalling pathways, activation of the stem cell/cancer-associated transcription factor *NANOG* and activation of the ligand-activated transcription factor *AHR*. Inhibited TFs included the tumour suppressing p53 and Rb pathways (including pathway members *RB1* and *CDKN2A* (p16)).

Western blot analysis of AHR in HMEC and vHMEC was performed (Additional file 1: Figure S3A). When active, AHR/ARNT form a complex, and this state was observed in vHMEC and not in HMEC (Additional file 1: Figure S3B). Intriguingly, *ANRT* was identified as inhibited in vHMEC by IPA, and the AHR/ARNT target genes *CYP1A1* and *CYP1B1* [25] are both down regulated at the mRNA level relative to HMEC (adjusted *P* = 0.03 and 0.000057, respectively). The predicted inhibition of *ARNT* and the apparent silencing of xenobiotic metabolising AHR target genes may suggest that activation of *AHR* in vHMEC is in relation to its putative roles in development or other novel processes rather than xenobiotic response.

The increased activity of *MYC* and a related pathway identified by IPA (Figure 2A and Additional file 2: Table S1) suggests a key role for activating *MYC* in the capacity of vHMEC to overcome senescence barriers. To investigate this further, we assessed the differential expression patterns of the MYC, PRC and CORE modules published by Kim *et al.* [26]. Together, these gene sets can distinguish the expression patterns of cancer cells from that of stem cells and are comprised of genes activated by *MYC* (MYC_MODULE) and genes silenced by PRC2 (PRC_MODULE) in cancer and stem cells and a set of genes activated in stem cells only by a core group of pluripotency factors (CORE_MODULE). In vHMEC, we observed enrichment of up- and down-regulated genes in the MYC and PRC modules, respectively (Figure 2C, D, respectively, family-wise error rate *P* value <0.05) and no significant change in the CORE module genes (*P* = 0.06, Additional file 1: Figure S2D), similar to the expression pattern of cancer [26]. Furthermore, the deregulation of the MYC and PRC modules matches the observed increase in *MYC* and *EZH2* expression and their predicted increase in activity by IPA.

### Tumour-suppressing miRNA are silenced in vHMEC

**Figure 3:**
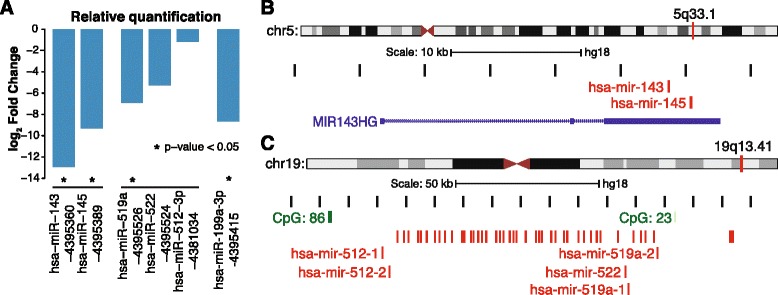
Differentially expressed miRNA in the HMEC model system. **(A)** Expression profile of the four differentially expressed miRNA (*hsa-mir-143, hsa-mir-145 hsa-mir-519a* and *hsa-mir-199a*), and *hsa-mir-519a* cluster associated miRNAs (*hsa-mir-522* and *hsa-mir-512*) in vHMEC. *hsa-mir-519a* clustered RNA exhibited a similar but not significant change in expression in vHMEC, indicating that this cluster may be regulated as a unit during carcinogenesis. **(B)** The *hsa-mir-143/145* cluster that exhibits simultaneous silencing in vHMEC. **(C)** The *hsa-mir-519a* cluster on chromosome 19q13.41 contains many other miRNA including two also assayed by the TLDA.

To assess the potential contribution of the deregulation of microRNA (miRNA) expression in the capacity of vHMEC to overcome senescence (and, therefore, in early carcinogenesis), we profiled miRNA expression in a subset of HMEC and early vHMEC (donors Bre12, Bre67 and Bre98) by Taqman® Low-Density Array (TLDA; Life Technologies, Carlsbad, USA). TLDA analysis identified four miRNA with a statistically significant (*P* < 0.05) loss of expression (Figure 3A). Notably, of the four statistically significantly differentially expressed miRNA, three fell into clusters (Figure 3B, C). Both *hsa-mir-143* and *hsa-mir-145* are encoded by the same gene, which accounts for their similar expression profile. Two copies of *hsa-mir-519a* are located within a very large cluster of miRNA on chromosome 19. Two other miRNA from the *hsa-mir-519a* cluster were assayed by the TLDA platform and exhibited a similar, although non-significant, change in gene expression (*hsa-mir-522* and *hsa-mir-512*, Figure 3A) indicating that this cluster may be regulated as a single unit.

Next, we identified which experimentally validated and predicted target genes of the four vHMEC-silenced miRNA in the TarBase and microrna.org databases [27,28] exhibited increased expression in vHMEC. This identified 470 interactions (196 unique genes, Additional file 2: Table S2) from the microrna.org database and six validated targets from the TarBase database (Additional file 2: Table S3). Notably, several key cancer-associated genes were included in the list of deregulated targets, including *MYC* (hsa-mir-145)*, EZH2* (hsa-mir199a) and members of the p53 pathway (*MDM2* and *CDKN1A* targeted by hsa-mir-199a and; hsa-mir-519a and hsa-mir-145, respectively). Additionally, hypergeometric testing using the MSigDB C2 Canonical Pathways database (Additional file 1: Figure S4) revealed that cell cycle regulation, proliferation and p53 regulated pathways were enriched within the predicted miRNA targets.

### Long-range epigenetic regulation is an early cancer event

**Figure 4:**
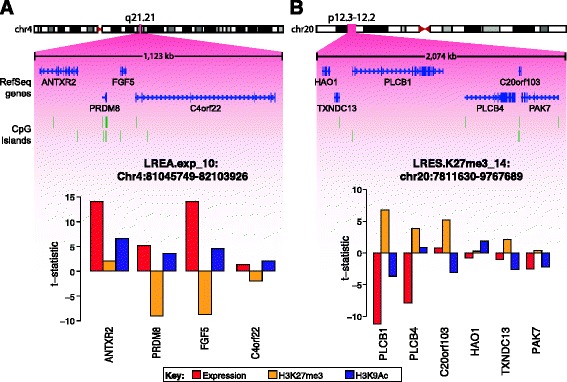
Example LRER regions occurring in vHMEC. **(A)** LREA region LREA.exp_10 at chromosome 4q21.21 was identified by its expression profile and contains four genes that exhibit concordant gain in expression, loss of H3K27me3 and gain in H3K9ac. **(B)** LRES region LRES.K27me3_14 (identified by gain in H3K27me3) at chromosome 20p12.3 contains six genes and exhibits concordant loss of expression and H3K9ac marking and gain in H3K27me3 marking.

While the deregulation of individual oncogenes and tumour suppressors is an important event in cancer, gene expression changes can also affect large domains of the genome containing multiple genes that exhibit a concordant change in expression in a phenomenon termed long-range epigenetic regulation (LRER) [29,30]. To study the role of long-range epigenetic activation (LREA) and silencing (LRES), we performed ChIP-chip analysis of H3K9ac on Bre12 and Bre38 and, H3K27me3 on Bre12, Bre38, Bre67 and Bre98 paired HMEC/vHMEC lines and combined with the expression data to look for concordant changes in chromatin-associated activation or repression. Analysis identified 33 regions of LREA and 41 regions of LRES based on change in expression, H3K9ac or H3K27me3 (Additional file 2: Table S4). On average, LREA regions were 0.5 Mb in length and contained an average of nine genes (Figure 4A) and LRES regions were 1.1 Mb and contained an average of eight genes (Figure 4B). Hypergeometric testing revealed that genes within LREA regions were enriched in gene sets associated with transcription and maintenance of the DNA (Additional file 1: Figure S5A), whereas LRES genes were enriched by gene sets associated with cancer and involved in the interaction with the extracellular environment (Additional file 1: Figure S5B). To investigate TF deregulation as a mechanism of LRER region formation, we interrogated LRER regions for TF binding sites (TFBS) associated with deregulated TF identified by IPA. TFBS were identified using a set of evolutionarily conserved, computationally derived TFBS available from the UCSC genome browser website [31,32]. The majority of TFBS tested exhibited no enrichment in LRER. However, LRES regions were significantly depleted for sites associated with AHR, ARNT, E2F family members and MYC (Additional file 1: Figure S6).

### DNA methylation changes occur early in carcinogenesis

**Figure 5:**
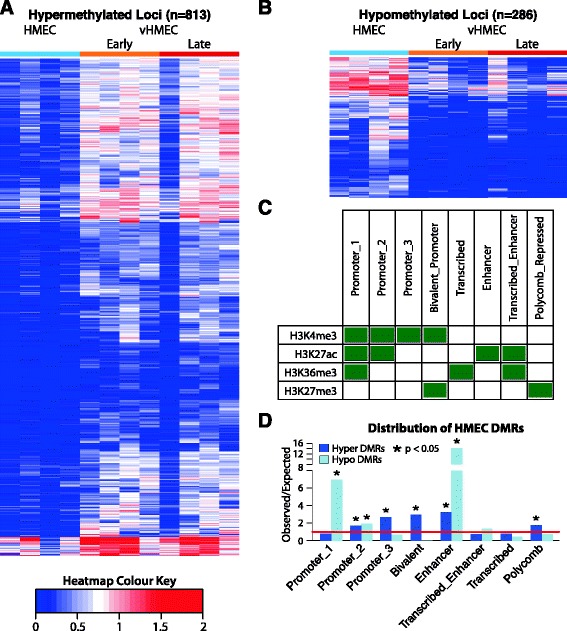
Summary of DNA methylation profiling of the HMEC model system. **(A)** The DNA methylation profile of the 813 identified DMRs in HMEC (light blue) and early and late vHMEC (orange and red, respectively). Loci hypermethylated in early vHMEC typically exhibit a similar profile in the late vHMEC time point despite extended time in culture. **(B)** A further 286 regions exhibited stable hypomethylation in the vHMEC samples. **(C)** ChIP-seq profiling was used to annotate the HMEC genome into eight categories based on the profile of H3K4me3, H3K27ac, H3K36me3 and H3K27me3 including four classes of promoter, regions of predicted transcriptional activity, putative enhancers and polycomb repressed loci. **(D)** Hypermethylated DMRs were frequently found at regions of predicted low activity such as Promoters 2 and 3, bivalent promoters and polycomb repressed loci as well as putative enhancers. Hypomethylated DMRs predominately affected regions of predicted high activity, such as promoters (Promoter_1) and putative enhancers.

The timing of DNA methylation changes during carcinogenic transformation remains largely unknown. To investigate the acquisition of differentially methylated regions (DMRs) during the earliest phases of carcinogenesis, we performed DNA methylation profiling using MBDCap-seq (see ‘Methods’) on HMEC lines (Bre12, Bre38, Bre67 and Bre98) and early and late passage vHMEC. Bioinformatic analysis identified 813 and 286 regions of hyper- and hypomethylation, respectively, in vHMEC (Figure 5A, B) that were stably maintained between the early and late vHMEC time points. Five gene-associated hypermethylated loci were selected for validation by clonal bisulphite sequencing and all regions exhibited an increase in the density of heterogeneous methylation between HMEC and vHMEC (Additional file 1: Figure S7).

Next, we asked if DMRs observed in vHMEC were associated with functional genomic features. Using RefSeq gene annotations, hypermethylated DMRs were statistically significantly (*P* < 0.05) enriched at CpG island loci and gene promoters (Additional file 1: Figure S8). To identify potential functional non-genic regions, we performed ChIP-seq profiling of H3K4me3, H3K27ac, H3K36me3 and H3K27me3 in Bre98 HMEC and vHMEC. Overlapping ChIP-seq peaks were used to classify 63,526 regions in HMEC and 61,096 in vHMEC into eight functional categories (designated Promoter_1, Promoter_2, Promoter_3, Bivalent_Promoter, Enhancer, Transcribed_Enhancer, Transcribed and Polycomb_Repressed, Figure 5C). Hypermethylated DMRs were enriched at *low* predicted activity promoters (Promoter_2 and 3), putative enhancer loci and polycomb regulated regions (Bivalent_promoter and Polycomb_Repressed). In contrast, hypomethylation was enriched at *high* activity promoters (Promoter_1) and *heavily* enriched (approximately 14-fold, *P* < 0.05) at the Enhancer category (Figure 5D).

Next we tested the influence of promoter and TFBS hypermethylation on the biology of vHMEC. A total of 272 hypermethylated DMRs were associated with the promoters of 161 genes. We used these genes to perform Gene Set Enrichment Analysis (GSEA) in the MSigDB C2 curated gene set database [33]. Promoter hypermethylated genes in vHMEC were enriched in 48 gene sets that were predominately associated with cancer, polycomb regulation and p53 (*n* = 20, 11 and 3 for cancer, polycomb and p53 associated gene sets, respectively, Additional file 1: Figure S9A). Interestingly, several individual p53 and polycomb-related genes were identified as deregulated at the expression level by IPA, suggesting that p53 and polycomb deregulation involves multiple levels of genomic control (that is, both the transcriptome and methylome). At TFBS, hypermethylated DMRs were enriched for several TFs, most notable of which were the ligand activated and cancer-associated AHR, the development-associated PAX5 and cell cycle/cancer-related E2F family members (Additional file 1: Figure S9B). Overall, vHMEC DMRs impacted an independent set of genes with similar functions to those identified by gene expression analysis (that is, p53, polycomb, E2F and AHR regulation).

### vHMEC differentially methylated regions are detected in breast cancer

**Figure 6:**
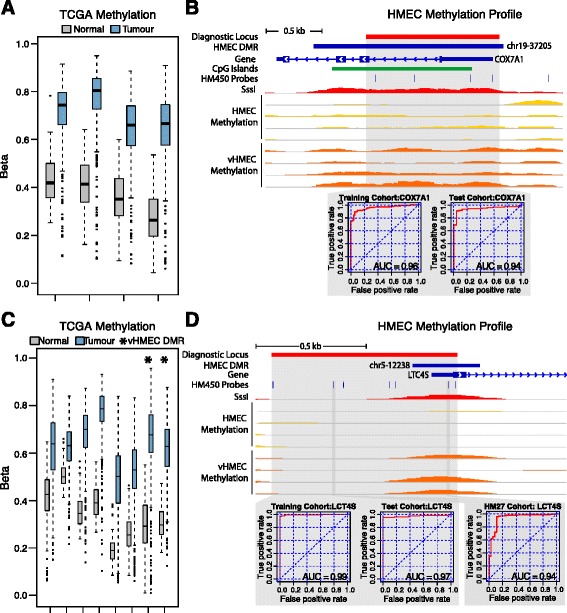
The methylation profile of vHMEC hypermethylated loci can identify tumour from normal in the TCGA breast cancer cohort. **(A)** HM450 probes overlapping with vHMEC DMR chr19-37205 are hypermethylated in tumours in the TCGA-BRCA cohort. **(B)** DMR chr19-37205 overlaps the *COX7A1* promoter associated CpG island. Methylation at the locus was able to separate tumour from normal tissue in the TCGA-BRCA cohort (AUC > 0.94). **(C)** vHMEC DMR chr5-12238 overlapped two HM450 probes; however, in the TCGA-BRCA cohort, several adjacent probes also exhibited increased methylation. **(D)** Hypermethylation of DMR chr5-12238 overlapping and adjacent probes (covering the *LCT4S* promoter) was specific to tumours in the TCGA-BRCA cohort (ROC analysis AUC > 0.97). Additionally, a small independent cohort of HM27 profiled samples also exhibited tumour-specific methylation at this locus (AUC = 0.94).

Differential methylation that occurs very early during transformation may provide useful biomarkers to detect breast cancer. To determine if the early DMRs we identified in vHMECs were present in breast cancer, we analysed Infinium HumanMethylation450 BeadChip® (HM450) DNA methylation data obtained from TCGA-BRCA cohort (see ‘Metho7ds’). Given the basal-like phenotype of vHMEC, we performed this analysis using all tumours and then separately with just the basal-like subtype. The 813 hypermethylated vHMEC DMRs overlapped with 2,430 HM450 probes, of which 1,560 or 961 were hypermethylated in all tumours or basal-like tumours, respectively (adjusted *P* values <0.05, Additional file 1: Figure S10A). This high frequency of hypermethylated probes in vHMEC DMRs represents an approximately twofold enrichment of hypermethylation over what is expected to occur at random (*P* < 0.05). The enrichment of vHMEC DMRs for TCGA-BRCA hypermethylation suggests that these regions may be able to distinguish cancer from normal breast cells in both a general and basal-like specific manner. Therefore, we performed a receiver operating characteristic (ROC) analysis on TCGA_BRCA methylation to identify regions of potential diagnostic significance across all breast cancer subtypes. From the 813 vHMEC DMRs, 38 could sensitively and specifically separate tumours from normal in the training cohort (Additional file 2: Table S5). In the test cohort, all 38 regions exhibited an area under the curve (AUC) of >0.91, indicating the analysis is robust. Further validation was performed for a subset of ten loci that also overlapped probes from the Infinium HumanMethylation27 BeadChip® (HM27) DNA methylation platform. In the HM27 cohort, these regions exhibited an AUC of between 0.76 and 0.98 (Additional file 2: Table S5), confirming their diagnostic capacity in an independent dataset. Examples of diagnostic DMRs, associated with *LCT4S* (promoter)*, COX7A1* (promoter), *TRIM62* (intronic) and *EPHB3* (promoter), are shown in Figure 6 and Additional file 1: Figure S11.

Given the early nature of methylation changes in vHMEC, it is possible that regions of hypermethylation are more extensive in cancer due to ‘spread’ of aberrant DNA methylation. Two candidate diagnostic vHMEC DMRS, associated with the genes *LCT4S* and *EPHB3*, exhibited a high AUC despite only overlapping two and three HM450 probes, respectively. In TCGA-BRCA cohort, several probes immediately adjacent to these regions also exhibited hypermethylation in cancer when compared to normal (Figure 6C and Additional file 1: Figure S11C) and could therefore improve the cancer specificity of these loci. With these additional probes included, ROC analysis revealed an increased AUC for both genes (Figure 6D AUC = 0.99 and Additional file 1: Figure S11D AUC = 0.99), supporting the possibility of methylation ‘spread’ in cancer. We also asked if DMRs were associated with altered gene expression in the same TCGA breast cancer samples. The correlation between methylation and expression was variable for each of the genes (*R*^2^ ≤ 0.11, 0.3, 0.23 and 0.6 for *LTC4S, TRIM62, EPHB3* and *COX7A1*, respectively); however, all diagnostic loci overlapped with the region where the correlation between methylation and expression was highest (Additional file 1: Figure S12), indicating that methylation at these loci may impact gene expression. Additionally, the DMRs in *EPHB3* and *TRIM62* overlapped with the binding sites of transcription factors identified as deregulated by IPA (Additional file 2: Table S6). Notably, the *EPHB3* methylated region, contained p53, E2F and AHR sites and the TRIM62 methylated region contained p53 and PAX5 sites, suggesting that these specific loci may be important in epigenetic deregulation of these genes.

**Figure 7:**
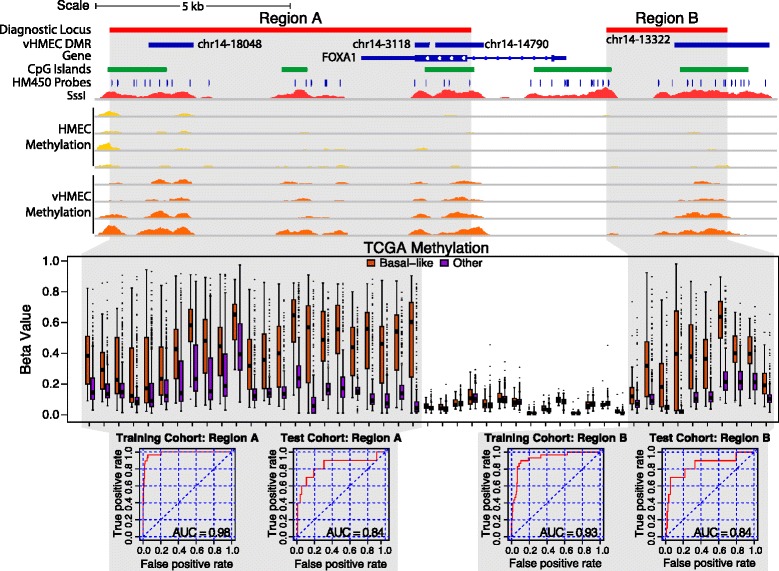
The FOXA1 locus is specifically methylated in basal-like tumours in the TCGA HM450 cohort. Two regions (regions A and B) containing four vHMEC DMRs that flank (but not cover) the *FOXA1* promoter exhibit increased methylation specific to basal-like tumours (region A AUC > 0.84 and region B AUC > 0.84).

In addition to general cancer diagnosis, vHMEC DMRs may be useful in separating basal-like tumours from the other subtypes. To this end, we repeated the ROC analysis of vHMEC DMRs to determine if any could separate basal-like tumours from all other tumour subtypes. Given the lower levels of methylation in basal-like tumours when compared to other subtypes (Additional file 1: Figure S10A), to reduce the identification of hypomethylated regions, we only analysed vHMEC DMRs that overlapped one or more HM450 probe that was hypermethylated in basal-like tumours (313 vHMEC DMRs overlapping 1,750 HM450 probes). Using an AUC cut off of >0.7, 14 candidate loci could separate basal-like from other tumours (Additional file 2: Table S7) although only four exhibited hypermethylation in basal-like when compared to other subtypes. For the four basal-like hypermethylated regions, two were associated with *FOXA1* (vHMEC DMRs chr14-3118 and chr14-14790), one with *LZTS1* (chr8-12256) and one within *CIRBP* (chr19-47460). We again inspected the regions for spread of hypermethylation into probes adjacent to the vHMEC DMR, and for the *FOXA1* and *LZTS1* regions, multiple adjacent probes did exhibit hypermethylation (Figure 7 and Additional file 1: Figure S13A, respectively) whereas *CIRBP* did not (Additional file 1: Figure S13B). *FOXA1* exhibited the most widespread methylation in basal-like tumours, with hypermethylation encompassing two separate regions; a very large region covering much of the *FOXA1* gene body and ending approximately 6 kb downstream of the transcription termination site (region A) and a shorter region approximately 2 kb upstream of the transcription start site (region B). ROC analysis revealed an AUC of 0.98 and 0.84 in the training and testing cohorts, respectively, for region A and 0.93 and 0.84 for region B (Figure 7). Similarly for *LZTS1* (Additional file 1: Figure S13A), with adjacent probes included, methylation was highly specific to basal-like tumours with AUC of 0.95 and 0.86 in the training and test cohorts, respectively. Hypermethylation of the *LZTS1* transcription start site did not exhibit an association with expression in TCGA breast tumours; however, *FOXA1* (*R*^2^ ≤ 0.75 and 0.67 for regions A and B, respectively) and *CIRBP* (*R*^2^ ≤ 0.212) both exhibited loss of expression with increased methylation (Additional file 1: Figure S14). Additionally, the *FOXA1* region A and *CIRBP* regions contained binding sites for p53 and AHR and, p53 and PAX5, respectively, all of which have been identified as deregulated in prior analyses (Additional file 2: Table S6).

## Discussion

The progression from a normal cell to a cancer cell involves genome-wide epigenetic deregulation and altered gene expression; however, the initial epigenetic lesions that occur during early carcinogenesis remain largely unknown. Here we utilised a model that recapitulates pre-malignant phase of basal-like breast carcinogenesis [34] and found significant alterations in DNA methylation and chromatin modification patterns that were associated with dysregulation of cancer-associated genes and pathways; specifically, genes and loci targeted by, *EZH2*/polycomb, *MYC*, *AHR* and the p53 pathway. Notably, these patterns of epigenetic deregulation were stable over an extended period of cell culture and occurred similarly in four independent HMEC lines, suggesting an orchestrated process of alteration in DNA methylation and chromatin modification.

The hypermethylation of key transcription factor binding sites occurs in vHMECs and may indicate a putative mechanism to establish genome-wide differential methylation in cancer. For example, activation of *AHR* may lead to a coordinated change in the epigenetic landscape by triggering the hypermethylation of its binding sites. *AHR* is a ligand activated transcription factor that has previously been implicated as having roles in differentiation and cancer [35-40] and we show that AHR is active in vHMEC. In MCF7 cells, *AHR* activation has been linked to the silencing of *BRCA1* by recruitment of DNA methyltransferases to the *BRCA1* promoter region [41-43]. Additionally, in keratinocytes *AHR* is reported to induce the silencing and hypermethylation of the *CDKN2A* (p16) tumour suppressor gene and the inhibition of p53 signalling [44]. Notably, the silencing of p16 and subsequent promoter hypermethylation is an essential component of the process of generating vHMEC [15,18]. Together, these findings may suggest that activation of *AHR* could contribute to the extensive epigenomic change observed in vHMEC (including hypermethylation of *CDKN2A*), with potentially far ranging effects on the biology of carcinogenesis, such as the inhibition of p53 in vHMEC [44]. The observed inhibition of p53 signalling in vHMEC is intriguing, given that vHMEC are reported to express high levels of wild type p53 protein [45]. However, instead of modulation or alteration of the p53 protein/gene, we observed DNA hypermethylation of its target genes and loci. Additionally, we identified loss of expression of the p53-interacting/tumour-suppressing miRNA *mir143/145* cluster. The *mir143/145* cluster silenced in vHMEC is reported to act downstream of p53 by promoting cell death [46], further suggesting that p53 activity is impeded via silencing of its targets and inhibition of downstream signalling. Similar to p53, the *PAX5* transcription factor also exhibited frequent hypermethylation of its target sites in vHMEC. Interestingly, *PAX5* itself is reported as being frequently methylated in breast cancer [47,48] and its overexpression in cell lines is reported to reduce the cancer phenotype by promoting normal epithelial characteristics [49]. In vHMEC, *PAX5* remains unmethylated, suggesting it is the disruption of *PAX5* targets rather than of the regulator itself that may facilitate carcinogenesis.

Interestingly, DMRs identified in vHMEC were extensively methylated in breast cancer, with hypermethylation frequently found to have extended into neighbouring regions. With their stability and very early occurrence, vHMEC hypermethylated loci could therefore serve as excellent biomarkers for the initial detection of cancer. Indeed, we found that a subset of the vHMEC DMRs exhibit a strong cancer-specific DNA methylation signature and additionally we determined a set of loci that could specifically identify basal-like tumours. Primarily, these loci were frequently associated with genes that have known roles in cancer, for example, *FOXA1, LZTS1* and *CIRBP*. Increased methylation at two regions surrounding *FOXA1* also exhibited a strong correlation with decreased expression. Notably hypermethylation of these two regions has previously been reported in leukaemia [50], colorectal cancer [51] and pancreatic cancer [52], indicating that they are potential regulatory loci for *FOXA1*. Given that basal-like cancers and HMEC express low levels of *FOXA1,* hypermethylation at these regions may play a role in reducing the transcriptional plasticity of the *FOXA1* locus. Interestingly, several candidate biomarker loci also overlapped with predicted binding sites of the p53, AHR and PAX5 transcription factors, further indicating a key role for hypermethylation at these transcription factor binding sites during carcinogenesis.

## Conclusions

The HMEC model system provides an excellent tool to study the biology of early breast carcinogenesis with the potential to deliver clinically relevant biomarkers for the detection and classification of breast cancer. Additionally, our results indicate that the initial cancer driving events involve genome-wide epigenetic silencing of transcription factor targets that potentially disrupt gene networks. Finally, we show that in pre-malignancy dysregulation of the epigenome is extensive and can occur across large domains with wide-ranging impact on the process of carcinogenesis.

## Methods

### Tissue culture

HMEC lines (Table 1) were generated from tissue removed from healthy women during breast reduction mammoplasty with informed consent and approval from the Sydney Adventist Hospital Human Research Ethics Committee and prepared according to the protocol set out in Stampfer *et al.* [53]. HMEC lines were expanded in MBD170 serum-free basal medium (Life Technologies, Carlsbad CA, USA) until proliferation slowed and the cells entered the selection phase (passage 4 to 7). Selection phase HMEC were maintained with twice-weekly media changes until large colonies (1 to 2 cm) of growth were observed (2 to 4 weeks). Growing cells were trypsinised and transferred to a new culture vessel, generating the vHMEC lines. At the time of surgery, all donors were aged between 23 and 43 years of age.

**Table 1:** **HMEC donor details**

**Donor**	**Age**	**Passage**		
		**HMEC**	**Early vHMEC**	**Late vHMEC**
Bre12	23	3* (3^#^)	10* (7^#^)	16* (14^#^)
Bre38	29	5* (3^#^)	11* (7^#^)	14* (26^#^)
Bre67	23	2* (2^#^)	7* (7^#^)	14* (14^#^)
Bre98	30	2* (2^#^)	8* (7^#^)	14* (14^#^)

*Passage used for expression profiling. ^#^Passage used for methylation profiling. HMEC, human mammary epithelial cells; vHMEC, variant HMEC.

### MBDCap-seq

Methylated DNA Binding Domain protein Capture followed by sequencing (MBDCap-seq) utilised 1 μg genomic DNA sonicated to mean fragment size of approximately 500 bp using a Bioruptor® water bath sonicator (Diagenode, Denville NJ, USA). Methylated DNA was captured using the Methylminer® Methylated DNA Enrichment Kit (Life Technologies, Carlsbad CA, USA) according to the manufacturer’s protocol and total captured DNA was eluted with one high salt wash (2,000 mM). Sequencing was performed on 10 ng MBD captured DNA on the Illumina GAIIX sequencing platform (Illumina, San Diego, CA, USA) at the Ramaciotti Centre for Gene Function Analysis (RCGFA) or The Beijing Genomics Institute (BGI) generating single end 36 bp reads at RCGFA and 50 bp at BGI.

Short sequence reads from Illumina sequencing were mapped to the human genome build HG18 with Bowtie [54]. Reads mapping to multiple locations, containing sequencing errors or duplicated reads were discarded from further analysis. The number of reads mapping at any given locus depends upon CpG density [55]. Therefore, to identify all genomic loci assayable by MBDCap-seq, an SssI treated 100% methylated sample (CpGenome Universally methylated DNA, Merck Millipore, Billerica MA, USA) was used to identify regions of the genome attracting a sufficient number of reads for the reliable detection of DNA methylation status. Specifically, the findPeaks function of the Homer peak-calling suite of programs [56] was applied to the SssI material (parameter settings of style = histone, size = 300, minDist = 300, tagThreshold = 18) which identified 230,655 assayable loci covering approximately 116 Mb across the genome. The edgeR Bioconductor package [57-59] was then used to model the distribution of reads in assayable loci to detect regions with statistically significant changes in read density.

### Clonal bisulphite sequencing

Bisulphite conversion of DNA was performed according to a previously published protocol [60]. Briefly, genomic DNA (1 μg) was incubated in cell lysis solution (100 ng/μl tRNA, 280 ng/μl Proteinase K, 1% SDS) at 37°C for 1 h to ensure complete protein digestion. NaOH (Sigma-Aldrich, St Louis, MO, USA) was added to a final concentration 0.3 M and DNA was denatured at 90°C for 5 min, then rapidly cooled on ice. The denatured sample was combined with a saturated solution of sodium metabisulphite (pH 5) and 10 mM Quinol (0.5 mM final Quinol concentration) and incubated at 55°C for 16 h. Bisulphite treated DNA was purified (Wizard® DNA Clean-Up System, Promega, Madison, WI, USA) according to the manufacturer’s protocol and desulphonated by the addition of NaOH to a final concentration of 0.3 M followed by incubation at 37°C for 15 min then purified by ethanol precipitation.

PCR amplification of bisulphite converted DNA was performed using Platinum Taq Polymerase (Life Technologies) according to the manufacturer’s protocol using the primers listed in Additional file 2: Table S8 under nested or fully nested conditions. PCR products were cloned into the pGEM T-easy cloning kit (Promega) and sequenced at Australian Cancer Research Foundation facility, located within the Garvan Institute of Medical Research, Sydney, Australia.

### Taqman low-density miRNA array

High-quality RNA was converted to cDNA using the Megaplex Pools® for microRNA expression analysis cDNA synthesis kit (Life Technologies, Carlsbad CA, USA) according to the manufacturer’s protocol, without pre-amplification. The total cDNA reaction was loaded onto a TLDA and qPCR was performed on an AbiPrism 7900HT (Life Technologies, Carlsbad CA, USA) sequence detection system. TLDA qPCR data was analysed using the HTqPCR R package [61] by the ΔΔCt method (normalised to the included control genes). Genes either lowly or invariably expressed across all patients and time points were excluded, leaving 116 out of 379 miRNA for further analysis. Predicted miRNA targets were identified using the microrna.org database [62]. To remove spurious interactions, predictions were limited to those that had a mirSVR score of < −0.5.

### Expression microarray

Gene expression profiling was performed by Affymetrix GeneChip® Human Gene 1.0 ST arrays (at one time point in the HMEC growth phase and two time points in the vHMEC phase referred to as early and late vHMEC, respectively), Taqman Low-Density miRNA arrays and RNA-seq.

RNA was extracted using TRIzol® reagent (Life Technologies, Carlsbad CA, USA) according to manufacturer’s protocol and quality assessed on the Agilent Bioanalyzer RNA Nano chip (Agilent Technologies, Santa Clara CA, USA) to ensure an RNA integrity number (RIN) of >9. RNA labelling and hybridisation to Affymetrix GeneChip® 1.0ST expression arrays was performed at the Ramaciotti Centre for Gene Function Analysis (UNSW, Sydney, Australia). Data analysis was performed using aroma.affymetrix [63]. Robust Multi-Array (RMA) normalisation to summarise gene-probe intensities and differentially expressed genes were identified using limma [64].

### TCGA data acquisition and analysis

Processed RNA-Seq expression data (level 3) and raw HM450 methylation data (level 1) were obtained from the TCGA data portal in January 2012 and clinical annotation from the 2012 TCGA-BRCA cohort primary publication [65]. Raw methylation data was pre-processed and background normalised with Bioconductor minfi package using preprocessIllumina (…, bg.correct = TRUE, normalize = ‘controls’, reference = 1) command. HM450 probes exhibiting differential methylation were identified using limma. Comparisons were carried out between all tumours and normal or basal-like tumours and all normal samples. For ROC analysis, the database was split randomly into two cohorts (75% and 25% of all samples, respectively) to allow training and testing of the models. ROC was performed using ROCR. For tumour-specific regions, regions exhibiting an AUC of >0.95 in the test cohort (after training) were selected as having potential utility as a diagnostic marker. For basal-like specific methylation, regions with an AUC of >0.7 were taken as having potential clinical utility.

### ChIP-seq

ChIP was performed according to the manufacturer’s protocol (17–295, Millipore, Billerica MA, USA). Briefly, approximately 2 × 10^6^ cells were fixed in culture medium plus 1% (*w*/*v*) formaldehyde for 10 min at 37°C. Fixed chromatin was sonicated to a mean fragment size of approximately 500 bp and immunoprecipitation performed for the chromatin marks H3K4me3, H3K27ac, H3K36me3 and H3K27me3 (see Additional file 2: Table S9 for antibody conditions). H3K4me3 profiling was performed on HMEC and early vHMEC from donors Bre67 and Bre98 and H3K27ac, H3K27me3 and H3K36me3 ChIP-seq was performed on donor Bre98 only. Illumina 36 bp single end sequencing was performed on 5 to 12 ng of ChIP DNA at either the Ramaciotti Centre for Gene Function Analysis or the Beijing Genomics Institute (see Additional file 2: Table S10 for sequencing summary statistics).

Short sequence reads from Illumina sequencing were aligned to the human genome build hg18 with bowtie [54] and regions of enrichment were identified using the Homer peak calling algorithm (Settings; H3K4me3: −minTagThreshold 10 -size 600 -minDist 600 -F 0 -L 0 -C 0, H3K27ac: −region -size 2000 -minDist 4000 -localSize 1000000 -F 0 -L 0 -C 0, H3K27me3 & H3K36me3; −region -fdr 1e-04 -minTagThreshold 40 -size 2000 -minDist 4000 -localSize 1000000 -F 0 -L 0 -C 0) [56]. Large regions of enrichment were broken into non-overlapping 1,000 bp tiles for the chromatin marks H3K27ac, H3K36me3 and H3K27me3 whereas, H3K4me3 exhibited smaller regions that did not need to be tiled. The density of sequence reads in regions of enrichment was assessed with edgeR [57-59] to establish regions of statistically significant chromatin marking in each time. Where regions had to be tiled, adjacent statistically significant regions were combined into a single region and an aggregate statistic calculated using Fisher’s exact test.

### ChIP-chip profiling

ChIP-chip profiling for H3K9ac was performed on donors Bre12 and Bre38, and H3K27me3 ChIP-chip was performed on donors Bre12, Bre38, Bre67 and Bre98.

ChIP and input DNA for array hybridisation was amplified to ensure sufficient yield prior to fragmentation and labelling (recommended 7.5 μg per ChIP-chip experiment). H3K27me3 ChIP DNA was amplified once with the GenomePlex® Complete Whole Genome Amplification (WGA) Kit (WGA2, Sigma-Aldrich, St. Louis, MO, USA). WGA2 amplification gave insufficient yield when performed on H3K9ac ChIP DNA. Therefore, H3K9ac ChIP DNA was amplified once with WGA2 and reamplified with the GenomePlex® WGA Reamplification Kit (WGA3, Sigma-Aldrich, St. Louis, MO, USA).

WGA material was cleaned up with the GeneChip® Sample Cleanup Module (Affymetrix, Santa Clara, CA, USA) and 7.5 μg was then fragmented and labelled for array hybridisation with the GeneChip® WT Double-Stranded Target Assay (Affymetrix, Santa Clara, CA, USA) according to the manufacturer’s protocols. Fragmented and labelled WGA ChIP and input DNA was hybridised to GeneChip® Human Promoter 1.0R arrays (Affymetrix, Santa Clara, CA, USA) at the RCGFA. Low level analysis and Model-based Analysis of Tiling arrays (MAT) normalisation of ChIP-chip data was performed using aroma.affymetrix [63]. Array signal across all promoters on the array was determined using Repitools [55,66,67]. ChIP data was then summarised to a *t*-statistic for a region of ±2 kb of all transcription start sites (TSS) with the function blocksStats [67]. Regions of LRER were identified by the methods set out in Bert *et al.* [30].

## Availability of supporting data

All sequencing and array data supporting the results of this article are available through Gene Expression Omnibus super series GSE58882.

## Additional files

Additional file 1: Supplementary Figures. Figures S1 to S14 and associated figure legends. Additional file 2: Supplementary Tables.
**Table S1** IPA TF deregulation predictions. **Table S2.** Predicted targets of deregulated miRNA (microrna.org). **Table S3.** TarBase validated targets of deregulated miRNA. **Table S4.** Coordinates and summary of vHMEC LRER. **Table S5.** Tumour specific vHMEC DMRs. **Table S6.** TFBS associated with candidate diagnostic DMRs. **Table S7.** Basal-like tumour specific vHMEC DMRs. **Table S8.** Bisulphite converted primer sequences, conditions and coordinates (HG18). **Table S9.** Antibodies used for ChIP-seq. **Table S10.** ChIP-seq validation primer sequences, conditions and coordinates (HG18).

## Abbreviations

AUC: area under the curve
ChIP: chromatin immunoprecipitation
ChIP-seq: ChIP followed by next generation sequencing
DMR: differentially methylated region
GSEA: gene set enrichment analysis
HM450: Infinium Human Methylation 450 beadchip
HMEC: human mammary epithelial cells
IPA: Ingenuity Pathway Analysis
LREA: long-range epigenetic activation
LRER: long-range epigenetic regulation
LRES: long-range epigenetic silencing
MBDCap-seq: Methylated DNA Binding Domain protein Methylated DNA Capture followed by next generation sequencing
MDS: multi-dimensional scaling
ROC: receiver operator characteristics
TCGA: The Cancer Genome Atlas
TCGA-BRCA: The Cancer Genome Atlas Invasive Breast Carcinoma Cohort
TF: transcription factor
TFBS: transcription factor binding site
TLDA: Taqman Low-Density Array
vHMEC: variant human mammary epithelial cells
