# Supplementary material for: Coordinated epigenetic remodelling of transcriptional networks occurs during early breast carcinogenesis
Source: Clin Epigenetics. 2015 May 1;7(1):52. doi: 10.1186/s13148-015-0086-0 (PMC4424562; doi:10.1186/s13148-015-0086-0)
Supplement: Additional file 1: Supplementary Figures. — Figures S1 to S14 and associated figure legends. [file 13148_2015_86_MOESM1_ESM.pdf]

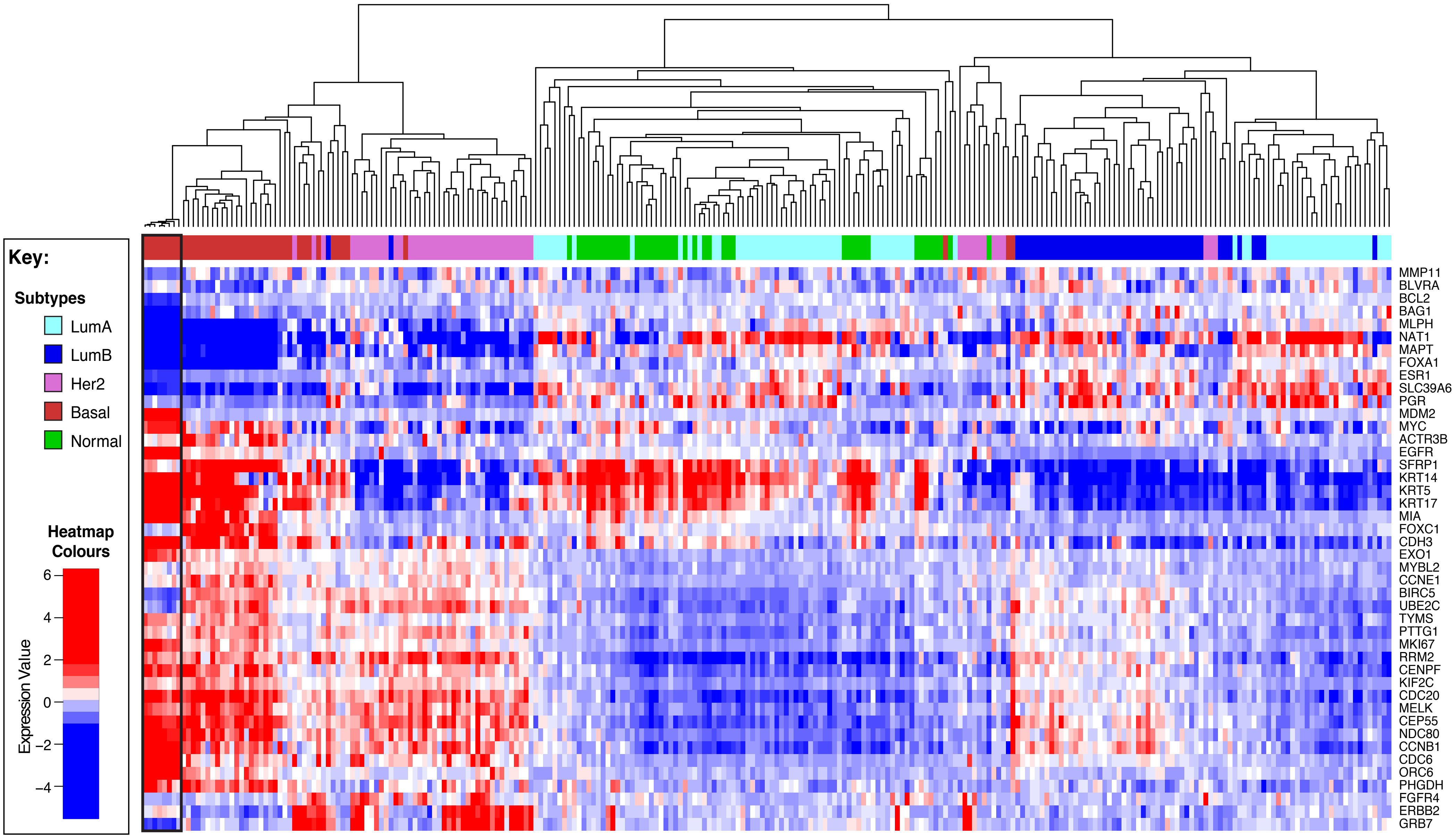


**Figure S1** PAM50 clustering of vHMEC in a second cancer cohort PAM50 clustering in a second breast cancer gene expression cohort [GEO:GSE3494] confirms the basal-like clustering pattern of vHMEC.

## Supp. Figure S2

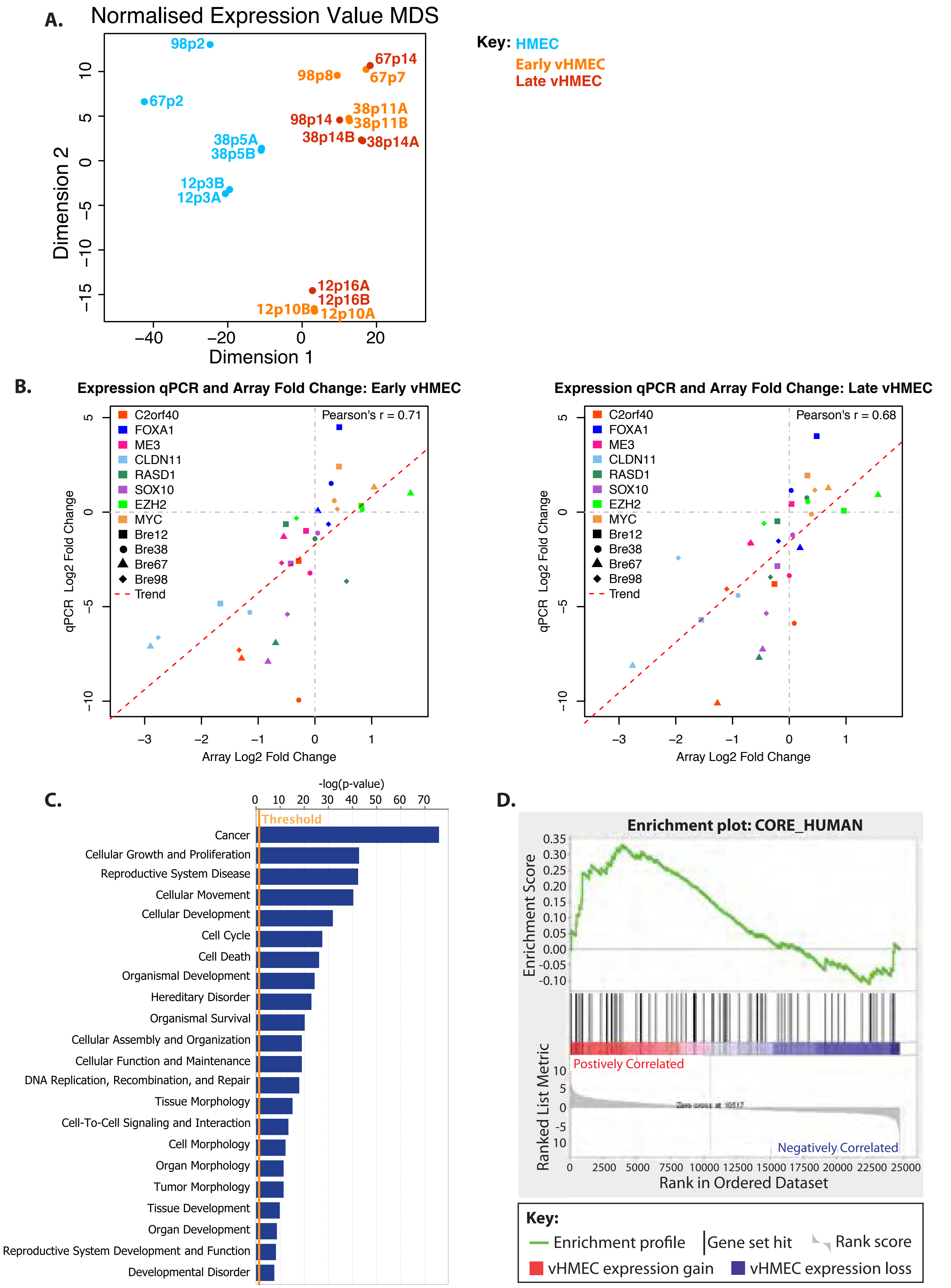

**Figure S2** Supplementary summary of HMEC gene expression profiling. (A) Multidimensional scaling analysis reveals separation of HMEC from all vHMEC samples on dimension one, with early and late vHMEC time points clustering close together, supporting the conclusion that the HMEC/vHMEC transformation is the largest source of variation in our dataset. (B) C2orf40, FOXA1, ME3, CLDN11, RASD1, SOX10, EZH2 and MYC differential expression was assayed by qPCR. Across all genes and donors, there was a high correlation between the expression difference as assayed by qPCR and expression arrays in both early and late vHMEC (Pearson's r = 0.71 and 0.68, respectively). (C) Pathways identified by IPA are most strongly enriched for biological processes relating to cancer and the cancer phenotype (for example, cell growth, cell cycle, cellular movement). (D) The Core pluripotency factor module (CORE module) that is only expressed in stem cells does not display enrichment for increased expression in vHMEC, supporting the cancer-like phenotype of vHMEC.

**Supp. Figure S3**

**A.**

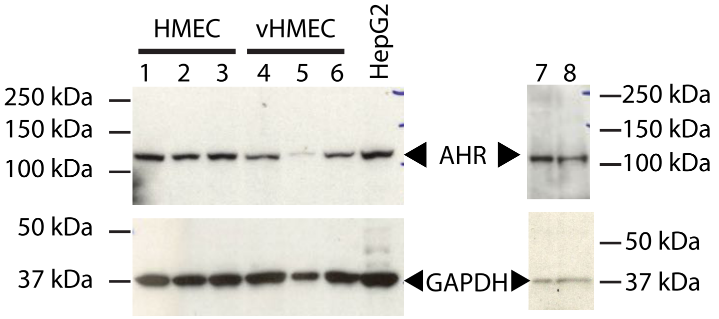


**Lane Order**

1. Bre12p4 Whole Cell Lysate
2. Bre38p4 Whole Cell Lysate
3. Bre98p4 Whole Cell Lysate
4. Bre12p10 Whole Cell Lysate
5. Bre38p10 Whole Cell Lysate
6. Bre98p9 Whole Cell Lysate
7. Bre78p4 Whole Cell Lysate
8. Bre78p9 Whole Cell Lysate

**B.**

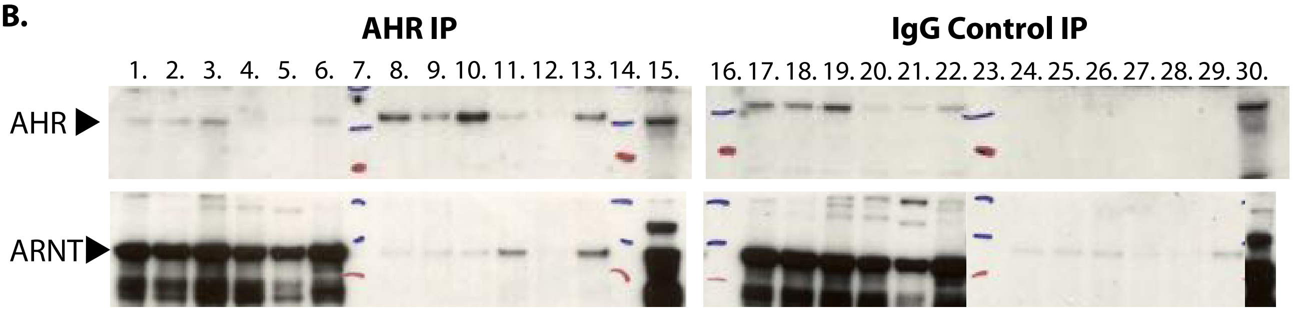


**Lane Order**

**AHR IP**

1. Bre38p4 AHR IP Unbound Protein
2. Bre78p4 AHR IP Unbound Protein
3. Bre98p4 AHR IP Unbound Protein
4. Bre38p10 AHR IP Unbound Protein
5. Bre78p9 AHR IP Unbound Protein
6. Bre98p9 AHR IP Unbound Protein
7. Ladder
8. Bre38p4 AHR IP Eluted Protein
9. Bre78p4 AHR IP Eluted Protein
10. Bre98p4 AHR IP Eluted Protein
11. Bre38p10 AHR IP Eluted Protein
12. Bre78p9 AHR IP Eluted Protein
13. Bre98p9 AHR IP Eluted Protein
14. Ladder
15. HepG2 Whole Cell Lysate (10ug)

**Non-specific IgG Control**

16. Ladder
17. Bre38p4 IgG IP Unbound Protein
18. Bre78p4 IgG IP Unbound Protein
19. Bre98p4 IgG IP Unbound Protein
20. Bre38p10 IgG IP Unbound Protein
21. Bre78p9 IgG IP Unbound Protein
22. Bre98p9 IgG IP Unbound Protein
23. Ladder
24. Bre38p4 IgG IP Eluted Protein
25. Bre78p4 IgG IP Eluted Protein
26. Bre98p4 IgG IP Eluted Protein
27. Bre38p10 IgG IP Eluted Protein
28. Bre78p9 IgG IP Eluted Protein
29. Bre98p9 IgG IP Eluted Protein
30. HepG2 Whole Cell Lysate (10ug)

**Figure S3** Western blot and Immunoprecipitation analysis of AHR in HMEC and vHMEC. (A) Western blot showing AHR protein levels in HMEC and vHMEC from donors Bre12, Bre38, Bre78 and Bre98. AHR protein levels appear similar or reduced in the four vHMEC strains. (B) AHR was precipitated from protein lysates of Bre12, Bre78 and Bre98 HMEC and vHMEC. Co-immunoprecipitation of ARNT with AHR was observed in vHMEC from donors Bre12 and Bre98.

**Supp. Figure S4**

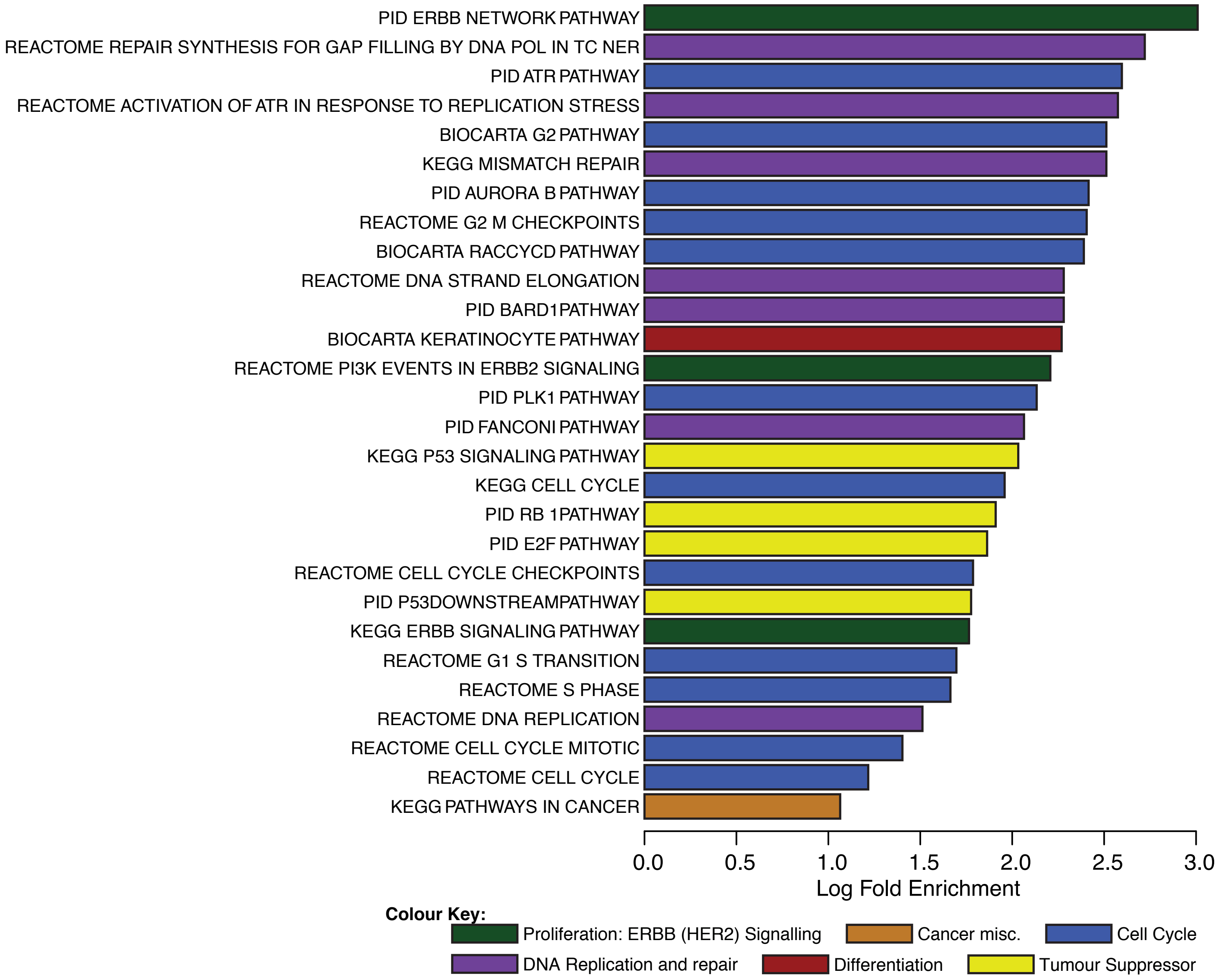


**Figure S4** Gene sets enriched for targets of differentially expressed miRNA Biological gene sets enriched for differentially expressed targets of hsa-mir143, hsa-mir-145, hsa-mir-199a and hsa-mir-519a. Enrichment of candidate miRNA deregulated genes in vHMEC was determined by hypergeometric testing. All gene sets identified are functionally related to the cancer phenotype (for example, proliferation, tumour suppression, differentiation and cell cycle).

**Supp. Figure S5**

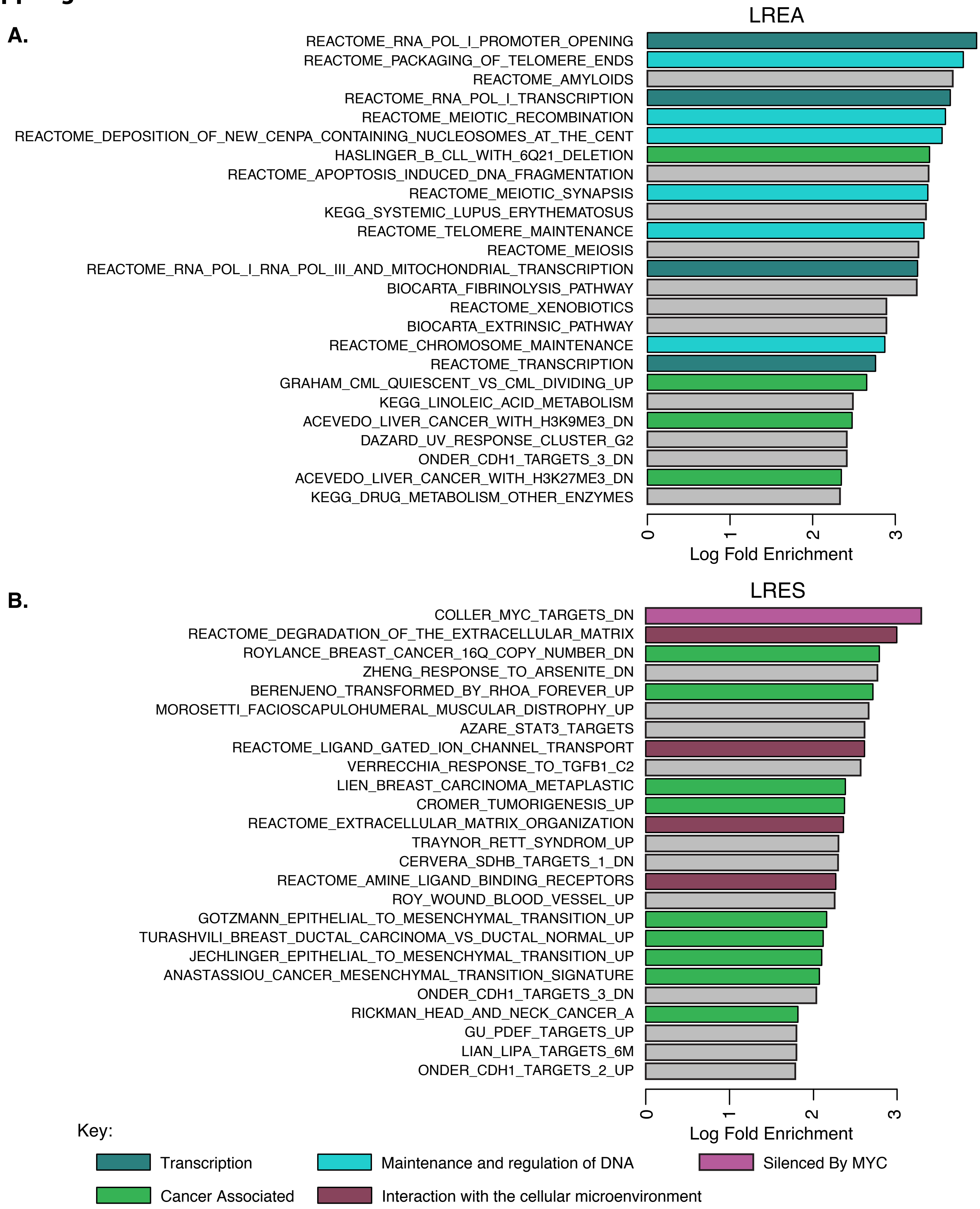


**Figure S5** Gene set enrichment of LRER genes. Hypergeometric testing of LRER genes in the MSigDB C2 database indicates that LRER may influence a wide range of cellular processes. LREA gene sets were dominated by those associated with the regulation of genomic function (transcription and genomic maintenance). LRES gene set enrichment was dominated by cancer-associated gene sets and gene sets involved in interaction with the extracellular environment. A set of genes suppressed by MYC was the most strongly enriched gene set for LRES genes.

**Supp. Figure S6**

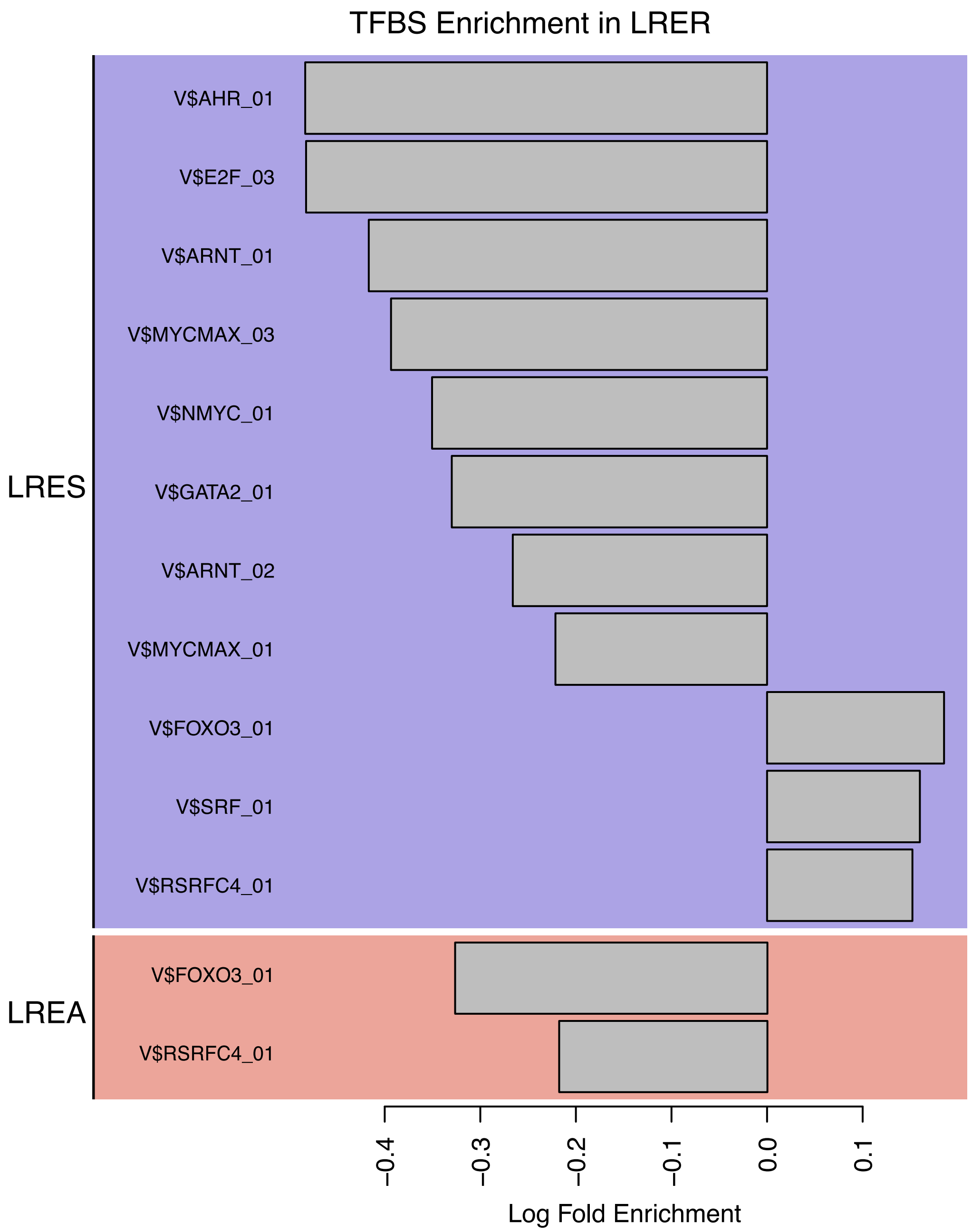


**Figure S6** Binding site enrichment of vHMEC deregulated TFs in LRER. Predicted TFBS for TF identified as deregulated by IPA were assessed for an association with LRER by hypergeometric testing. The bulk of TF exhibited no relationship with LRER. However, TFBS belonging to AHD, ARNT, MYC and E2F family members were all depleted within LRES regions, indicating these factors do not influence LRER.

# Supp. Figure S7

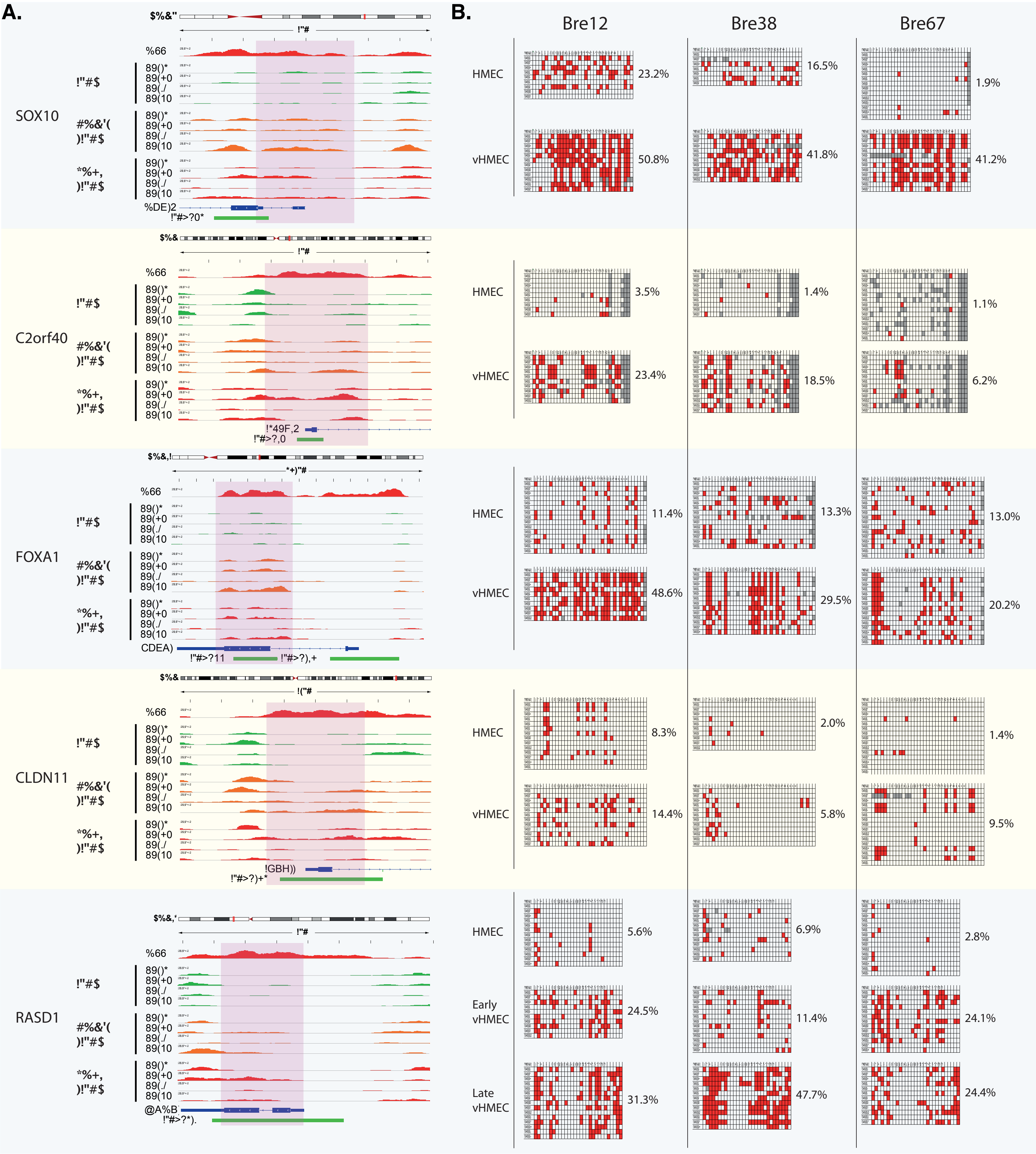


**Figure S7** Validation of MBDCap-seq by clonal bisulphite sequencing. (A) Genome browser images displaying DNA methylation levels as assayed by MBDCap-seq in HMEC (green), early vHMEC (orange) and late vHMEC (red). The SSSI track is 100% methylated and represents maximal possible signal at a given locus. The methylation profile of SOX10, C2orf40 (a.k.a Oesophageal Cancer Related Gene 4), FOXA1, CLDN11, and RASD1 in HMEC and vHMEC by MBDCap-seq matches the observed methylation levels as assayed by (B) clonal bisulphite sequencing (1 row/clone, 1 column/CpG site, red boxes are methylated sites and white boxes are unmethylated. Grey boxes represent sites where the sequencing base call was unreliable).

## Supp. Figure S8

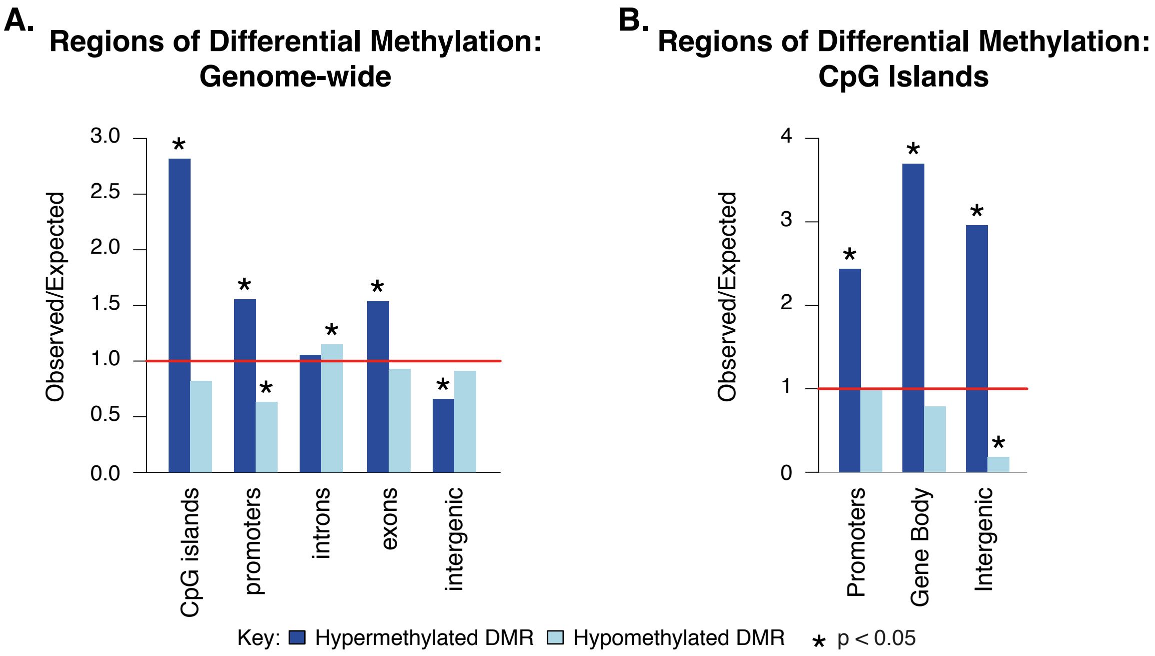


**Figure S8** Distribution of vHMEC DMRs relative to RefSeq annotations and CpG islands. (A) Hypermethylated DMRS are enriched at loci associated with CpG islands and transcription (promoters/exons) whereas hypomethylated DMRs are enriched at introns and depleted at promoters. (B) CpG island hypermethylation is enriched irrespective of genomic location (promoter, gene or intergenic), hypomethylation is not associated with CpG islands, even at intergenic loci.

## Supp. Figure S9

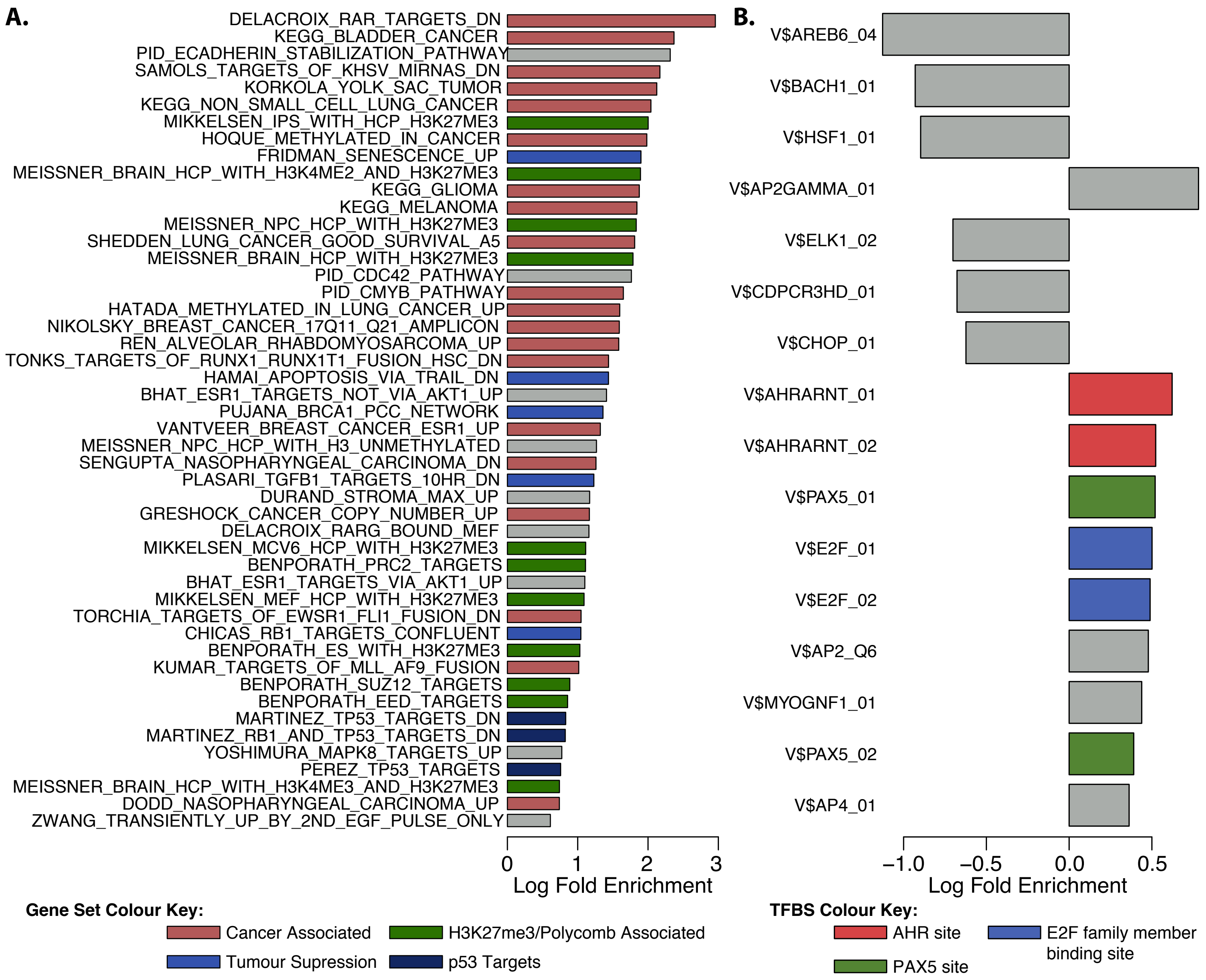


**Figure S9** Enrichment of hypermethylation in functional gene sets and at transcription factor binding sites. (A) Hypermethylated DMRs impact a wide range of biological processes, many of which are associated with known cancer pathways (for example, p53 and polycomb regulation). (B) Hypermethylation was enriched at sites associated with approximately 6 TF. Interestingly, AHRARNT sites are the binding location of a complex of AHR and ARNT, both of which were identified as deregulated by IPA. Other cancer-related factors include TFAP2C, PAX5 and the cell cycle regulating E2F family.

## Supp. Figure S10

**A.**

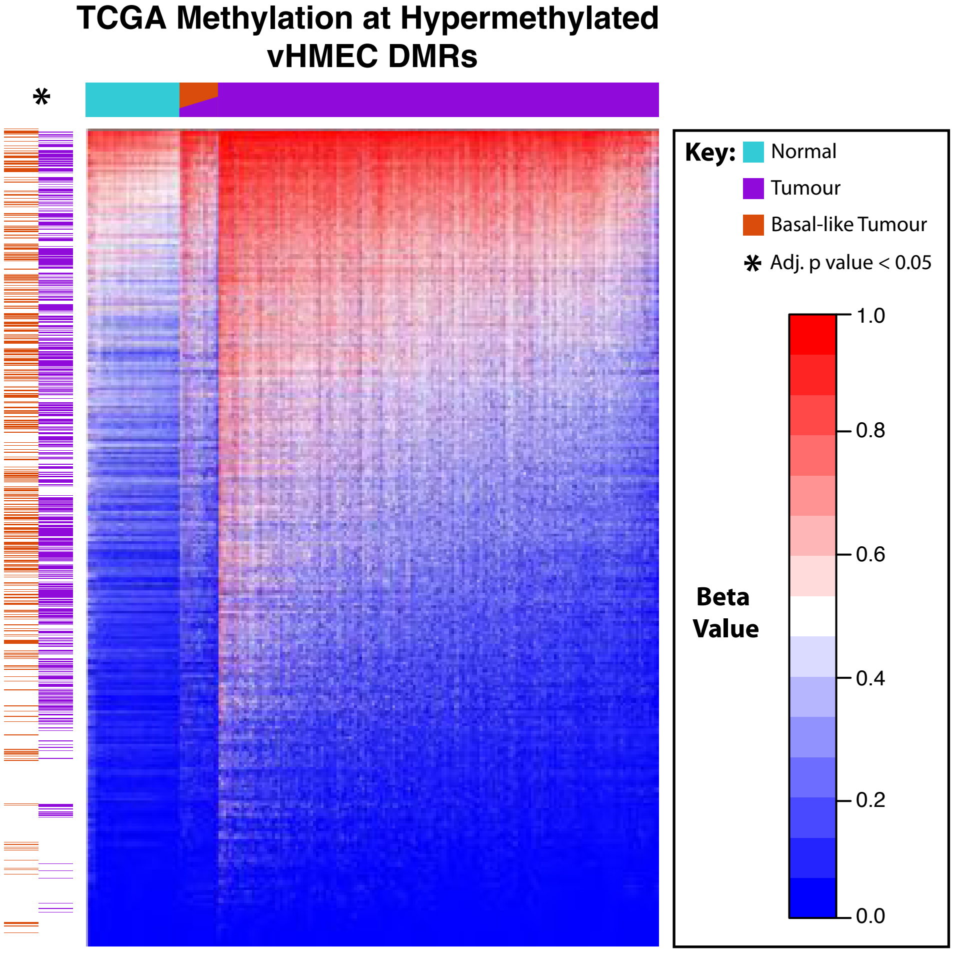


**B.**

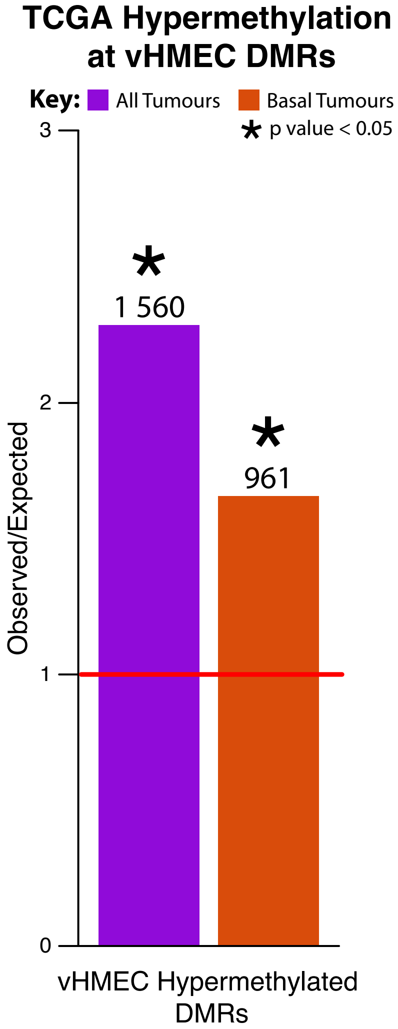


**Figure S10** TCGA-BRCA cohort methylation at vHMEC hypermethylated DMR loci. (A) Methylation levels were higher in Basal-like or all other tumours in the TCGA-BRCA cohort when compared to normal. This difference was frequently statistically significant ($P < 0.05$, see y axis). (B) Statistically significant hypermethylation in the TCGA-BRCA cohort at HM450 probes overlapping vHMEC hypermethylated loci occurred approximately twofold more frequently than is expected to occur at random ($P < 0.05$), in all tumours and in the basal-like subset.

**Supp. Figure S11**

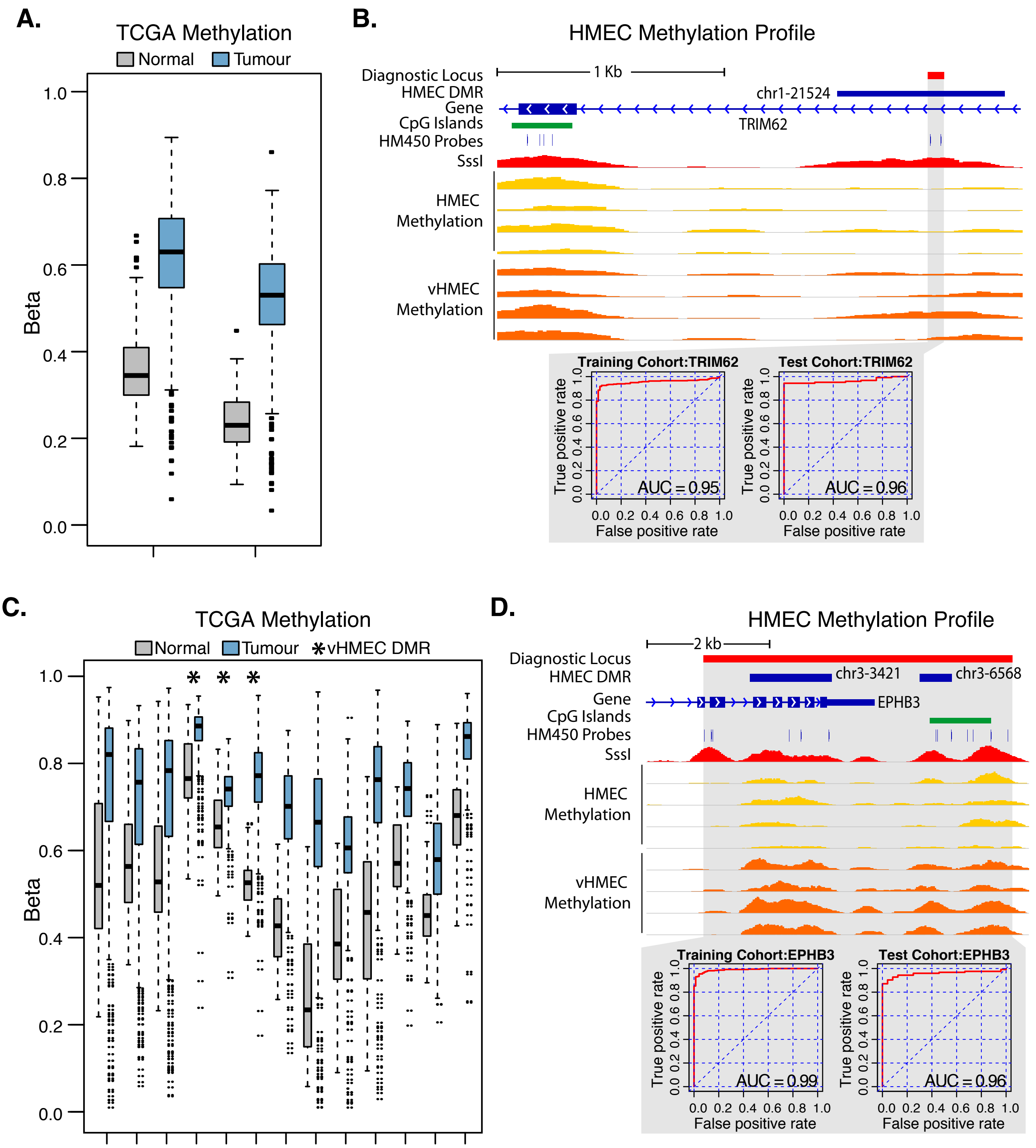


**Figure S11** TRIM62 and EPHB3 methylation is cancer specific. (A) Methylation levels in the TCGA-BRCA cohort of HM450 probes overlapping the TRIM62 region are increased in tumours when compared to normal. (B) Hypermethylation of an intronic region of the TRIM62 gene was specific to tumours by ROC analysis (AUC > 0.95). (C) HM450 probes overlapping the EPHB3 5′ end are hypermethylated in tumours in the TCGA-BRCA cohort. (D) Hypermethylation of a large region of the EPHB3 5′ end encompassing two vHMEC DMRs was also specific to tumours (AUC > 0.96).

## Supp. Figure S12

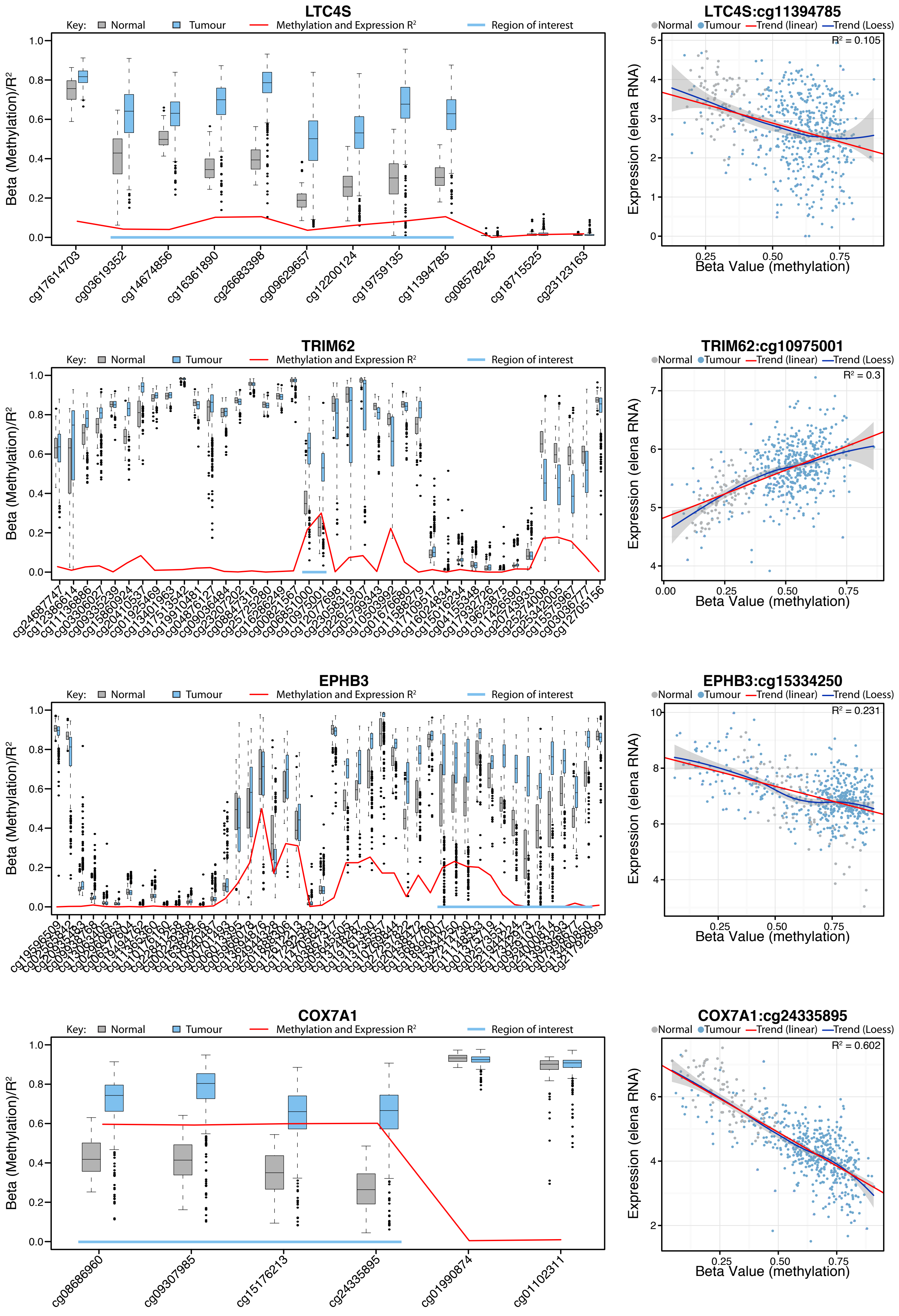

**Figure S12** Expression and methylation at candidate tumour-specific biomarker loci correlated in the TCGA cohort. The expression/methylation correlation observed was variable across all HM450 probes in candidate biomarker loci and the surrounding regions. Where the strongest correlation was observed always overlapped (at least partially) with the biomarker loci. A scatter plot of methylation and expression for one example probe for each of the biomarker loci is displayed in the right hand panel.

## Supp. Figure S13

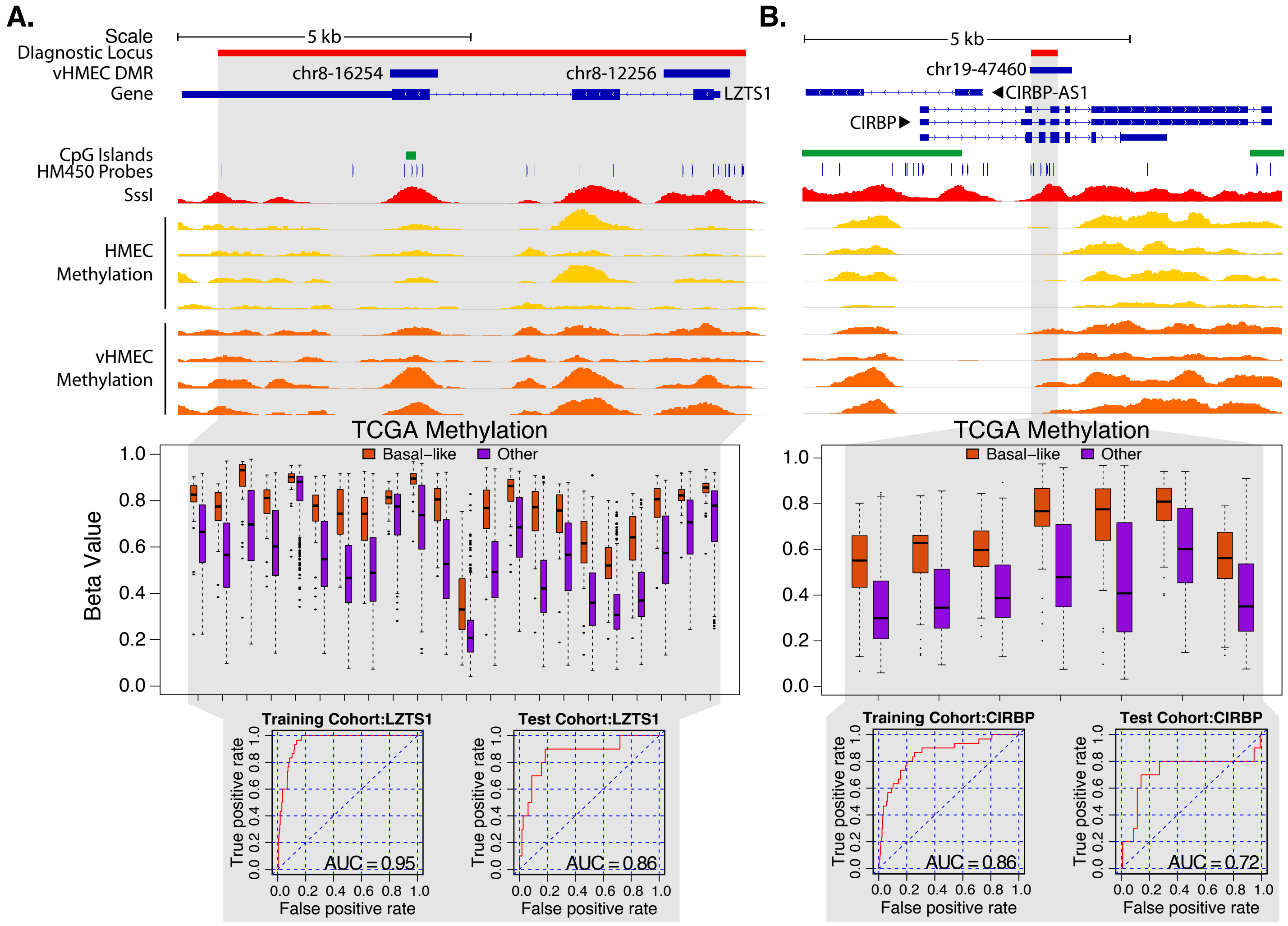


**Figure S13** Basal Cancer-specific methylation of LZTS1 and CIRBP. (A) A region spanning most of the promoter and body of LZTS1 and containing two vHMEC DMRs exhibits basal-like specific hypermethylation by ROC analysis (AUC > 0.86). (B) A small intragenic region of CIRBP is hyper-methylated in vHMEC and exhibits basal-like tumour-specific methylation in the TCGA cohort (AUC > 0.72).

# Supp. Figure S14

A.

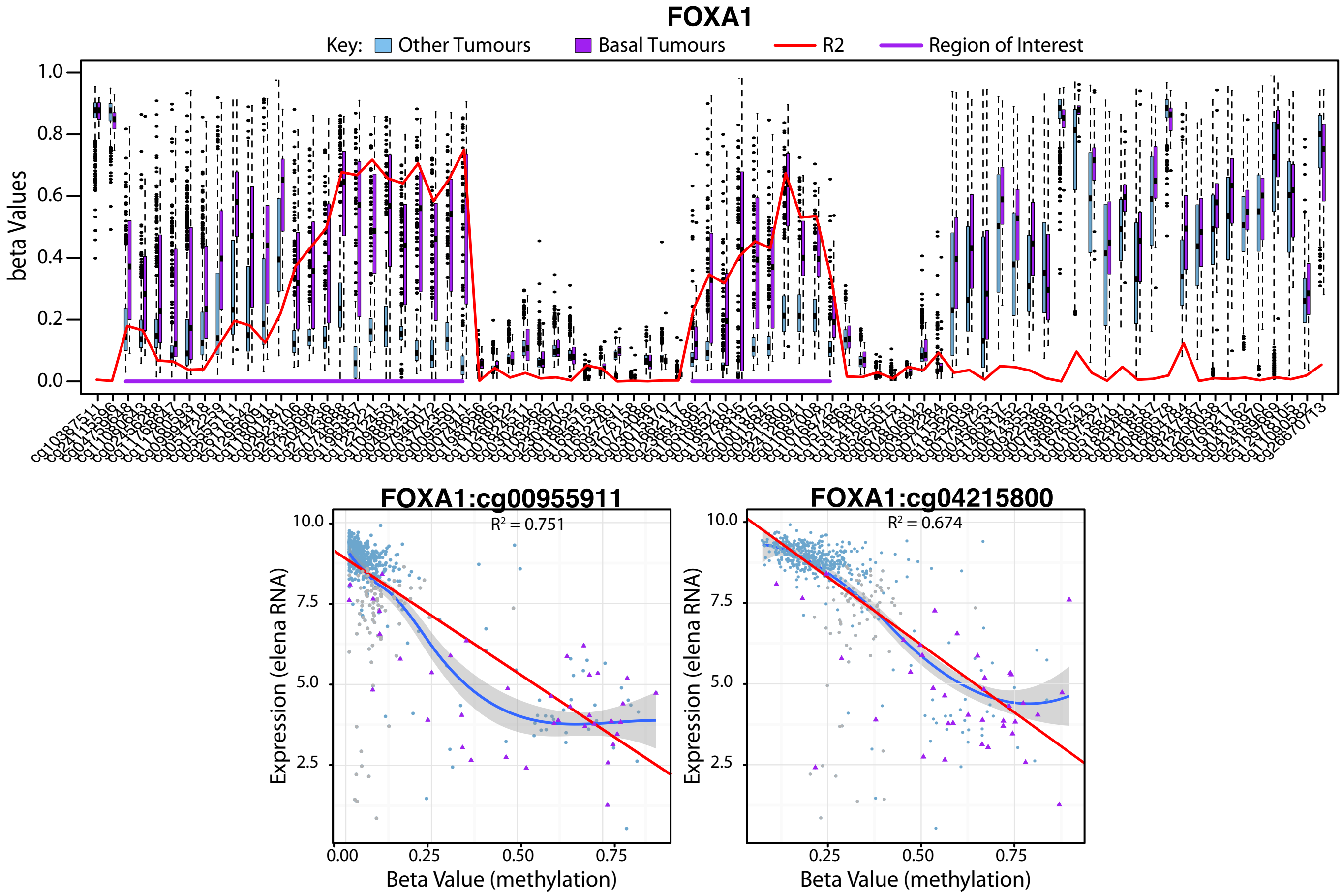


B.

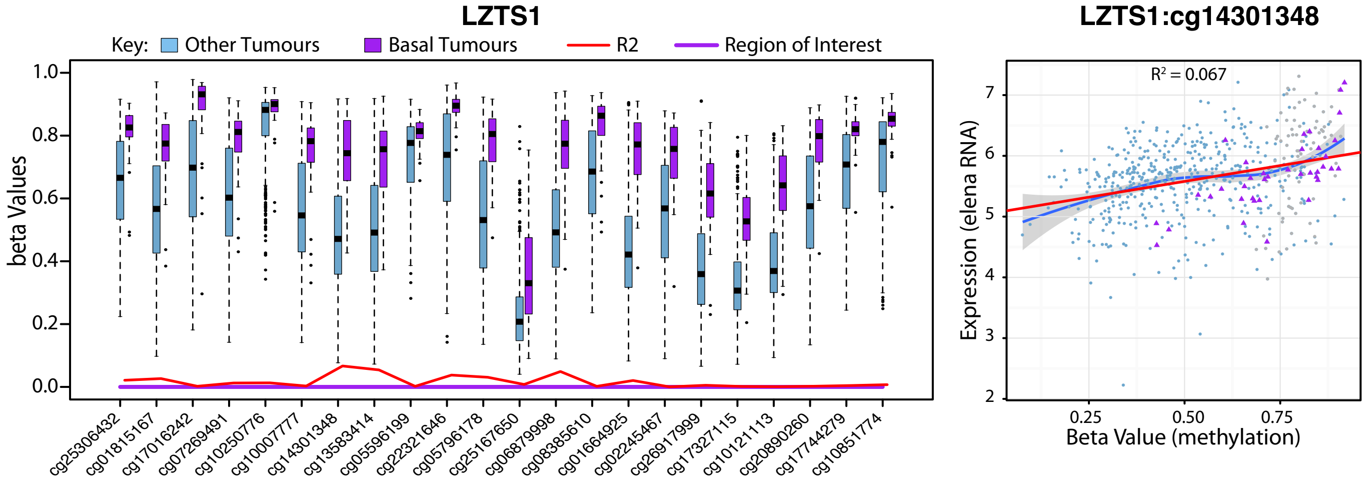


C.

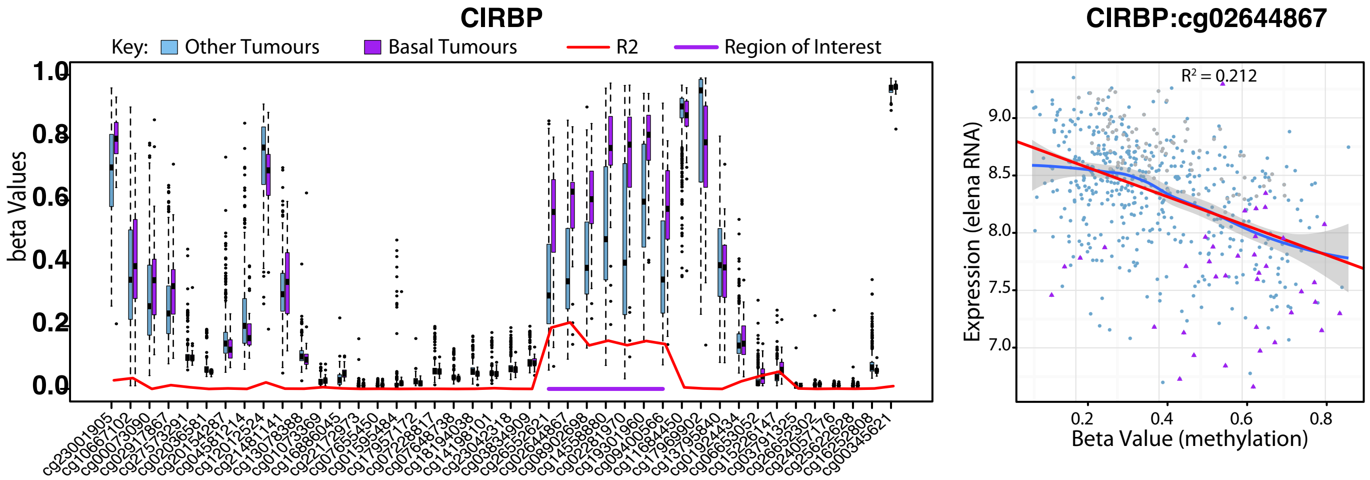

**Figure S14** Expression and methylation at candidate basal-like tumour-specific biomarker loci correlated in the TCGA cohort. The expression/methylation correlation observed was variable across all HM450 probes in candidate biomarker loci and the surrounding regions. Where the strongest correlation was observed always overlapped (at least partially) with the biomarker loci. This was particularly strong for the two FOXA1 association loci. A scatter plot of methylation and expression for one example probe for each of the biomarker loci is displayed in the right hand panel.
